# Gut Virome and Aging: Phage-Driven Microbial Stability and Immune Modulation

**DOI:** 10.14336/AD.2025.0854

**Published:** 2026-09-04

**Authors:** Kaleem Ullah, Rabia Kanwar, Saqib Ali, Saif Uddin, Izza Izza, Irfan Ahmad, Khalid J Alzahrani, Abdul Qadeer, Chun-Ting Chu, Chien-Chin Chen

**Affiliations:** ^1^Directorate General (Research) Livestock & Dairy Development Department Peshawar, 25000, Khyber Pakhtunkhwa, Pakistan.; ^2^Institute of Microbiology, University of Agriculture Faisalabad, 38000, Punjab, Pakistan.; ^3^Huazhong Agricultural University, Wuhan, Hubei, China.; ^4^University of Veterinary and Animal Sciences Lahore, Punjab, Pakistan.; ^5^Department of Clinical Laboratory Sciences, College of Applied Medical Sciences, King Khalid University, Abha, Saudi Arabia.; ^6^Health and Medical Research Center, King Khalid University, AlQura'a, Abha, Saudi Arabia.; ^7^Department of Clinical Laboratories Sciences, College of Applied Medical Sciences, Taif University, Saudi Arabia.; ^8^Department of Cell Biology, School of Life Sciences, Central South University, Changsha, China.; ^9^Division of Colon and Rectal Surgery, Department of Surgery, Ditmanson Medical Foundation, Chia-Yi Christian Hospital, Chiayi 600, Taiwan.; ^10^Department of Pathology, Ditmanson Medical Foundation Chia-Yi Christian Hospital, Chiayi 600, Taiwan.; ^11^Doctoral Program in Translational Medicine and Rong Hsing Translational Medicine Research Center, National Chung Hsing University, Taichung 402, Taiwan.; ^12^Department of Cosmetic Science, Chia Nan University of Pharmacy and Science, Tainan 717, Taiwan.; ^13^Department of Biotechnology and Bioindustry Sciences, College of Bioscience and Biotechnology, National Cheng Kung University, Tainan, Taiwan.

**Keywords:** Gut, Virome, Dysbiosis, Phage, Microbiome

## Abstract

The gut harbors trillions of microbes that play essential roles in metabolism and immunological functions. The virome, an important but understudied component of the gut microbiota, comprises bacteriophages commonly referred to as bacterial viruses, among other inhabitants. Understanding the interplay between the gut virome and bacteria can illuminate the dynamics of microorganisms in both healthy and diseased states. This knowledge can open new avenues for treatment, such as phage therapy and microbiota modulation, which aim to restore balance in the gut and improve overall health. The composition and diversity of the gut microbiome and virome undergo significant changes, which have been linked to increased susceptibility to infections, chronic inflammation, and age-related disease. Exploring the gut virome’s role in aging may provide insights into how viral and bacterial interactions influence immune senescence and gut resilience in the elderly population. This review provides an overview of the current understanding of the relationship between the gut virome, specifically phages associated with gut bacteria, and mechanisms of phage-host interactions, shedding light on how these factors affect and influence communities. Additionally, this review also explores the impact of the gut virome on gut resilience and its role in shaping and influencing the host’s immune response. Maintaining a healthy gut requires a stable and robust virome. Therefore, future research on the virome is crucial for the development of microbe-based products that enhance human and animal health and aid in addressing specific diseases.

## 1. Introduction

The importance of microbiota for human health and well-being has been increasingly recognized over the years. Research has demonstrated that these microbial residents play vital roles, such as aiding digestion, strengthening the immune system, and protecting against harmful pathogens [1]. Advances in molecular techniques have improved our understanding of the complex interactions between humans and microbes, revealing meaningful links between changes in the microbial ecosystem and conditions like dysbiosis. These developments have also highlighted the diverse range of viruses associated with the gut microbiota. The term “human gut virome” refers to the entire community of viruses or virus-like particles (VLPs) associated with the gut microbiota. Notably, the gut virome is mainly composed of prokaryotic viruses (bacteriophages or phages), reflecting the dominance of bacterial communities within the gut microbiota [2, 3]

The International Committee on Taxonomy of Viruses (ICTV) currently recognizes nineteen families of phages that infect bacteria or archaea, of which only two-*Inoviridae* and *Microvirida*e possess single-stranded DNA genomes. The distribution, genetic characteristics, and ecological roles of Microviridae remain largely unexplored [4]. Historically, research has primarily focused on the bacterial microbiome [5]. However, recent advances in metagenomics have led to the identification and characterization of an increasing number of native viral particles within these communities. The digestive systems of animals and humans, from the oral cavities to the intestines and excretion, have become areas of significant interest. Advanced sequencing and computational analyses have revealed a broad diversity of viromes in companion animals such as dogs and cats, as well as in farm animals and wildlife, including bats [6]. Recent studies have identified members of the Microviridae family in water and the intestines of both animals and humans, suggesting potential ecological implications [7, 8]. This family comprises two ICTV-approved subfamilies, Bullavirinae and Gokushovirinae, along with two provisional groups, Pichovirinae and Alpavirinae, which the ICTV has not yet officially recognized [9]. The Bullavirinae subfamily includes three genera: Alpha3microvirus, G4microvirus, and Phix174 microvirus, which infect enterobacteria, with the well-studied bacteriophage Phix174 serving as the family archetype. The Gokushovirinae subfamily is known as the target of obligate intracellular parasites from the genera *Chlamydia*, *Bdellovibrio*, and *Spiroplasma* [10].

The intestinal virome includes bacteriophages (commonly known as phages) and eukaryotic viruses. The human gut virome includes all viruses and virus-like particles (VLPs) linked to the gut microbial community. It mainly consists of prokaryotic viruses, reflecting the predominance of bacteria in the gut microbiome [11]. Phages in the human gut are believed to be present in quantities similar to their bacterial hosts. Typically, bacteria are found at about 10^9^ per gram of feces, while VLPs range from approximately 10^8^ to 10^9^ per gram. Most virus-like particles (VLPs) come from bacteriophages [12].

Maintaining balance within the complex gut microbial ecosystem is crucial for supporting the host's health and preventing disease development [13]. Conditions such as diabetes and Crohn's disease are linked to changes in the gut's commensal flora, a phenomenon called dysbiosis [14]. Dysbiosis leads to fluctuations in both gut microbiota and phage activities [15]. Recent studies emphasize the impact of the virome, especially phages, on the development of various medical conditions, including gastrointestinal disorders and cancer [16]. Acute diarrhea is a leading cause of death worldwide, especially among children, with viruses being major contributors. About 40% of diarrheal cases have unknown causes. The advent of metagenomic sequencing has enabled the discovery of new viruses that may be responsible for these illnesses [17]. Advances in omics technologies now allow comprehensive analysis of the entire animal virome, enhancing understanding of its composition, functions, and effects on animal health and disease [18]. Combining omics techniques with bioinformatics has helped researchers use microbiome profiling in clinical settings and identify underlying causes of disease. Studying microbiome changes related to disease is essential for pinpointing causes and understanding how microbiomes influence disease progression. Gaining insight into how microbiome imbalances contribute to disease development can lead to new strategies for disease control and improved treatment outcomes [19]. Some research on yak diarrhea shows that changes in the gut microbiota of adult yaks with diarrhea may facilitate pathogen colonization and spread within the gastrointestinal tract [13]. Similarly, Alsaaod and colleagues observed microbiota shifts during bovine digital dermatitis, identifying treponemes and Porphyromonas levii as potential causes of persistent claw horn lesions in cattle [14].

In addition to their links with various diseases, the gut virome plays a vital role in the health of both animals and humans. Animal viromes inhabit mucosal surfaces and remain throughout the body even after viral infections are cleared. Like the bacteriome, the virome influences the host's immune system, playing a key role in maintaining balance and controlling inflammation on mucosal surfaces [15]. The virome produces viruses that modulate the host's immune response, triggering immune reactions without causing clinical illness [16]. The functional diversity of the human gut virome is currently being studied, revealing a wide range of functions. Recent metagenomic studies have shown that gut phages carry genes mostly beneficial for intestinal bacteria. These genes help bacteria adapt to their environment, are linked to bacterial virulence, and support the stability and resilience of the host's microbiome [17]. Understanding the dynamics of the gut virome, especially bacteriophages, is an emerging area in aging research. Although the bacterial part of the microbiome has been extensively studied, the role of the virome, particularly in maintaining microbial balance and influencing immune responses during aging, remains less explored. Given the increasing recognition of phage-host interactions as key regulators of microbial ecology, we suggest that age-related changes in the gut virome could impact host health by altering microbial composition, immune responses, and mucosal resilience.

The growing field of gut microbiome research has thoroughly examined bacterial changes that occur with age, but the function of the virome, especially the phageome, is still intriguing and not well known. Contemporary research frequently provides descriptive inventories of age-related changes in viral diversity and composition [12, 18], but a comprehensive synthesis of these findings is absent. A central debate concerns whether these viromic alterations are catalysts or outcomes of aging do variations in phage dynamics (e.g., heightened temperate phage activity) directly facilitate microbial instability and immunosenescence, or are they merely consequences of age-associated immune deterioration and bacterial dysbiosis [19, 20] Moreover, the interpretation of these findings is impeded by methodological obstacles, such as database biases favoring temperate phages and the complexities of delineating host-virus interactions in vivo [21]. This review transcends mere description to critically evaluate current information, concentrating on the potential for phage-mediated control of the gut microbiome and immune system in the context of aging. We will clearly point out these main disagreements, talk about the methodological problems that make it hard to understand, and find important knowledge gaps that need to be filled in order to use the virome as a possible treatment target for age-related decline.

This review aims to compile current knowledge about the aging gut virome, with a focus on phage-driven mechanisms, while highlighting important gaps and directions for future research. This work critically reviews both human and relevant animal studies, emphasizing the virome as a potential target for future diagnostics and treatments designed to promote healthy aging and reduce age-related diseases.

## 2. Compositional changes in the gut virome with age

### 2.1. Human virome

**Figure 1.**
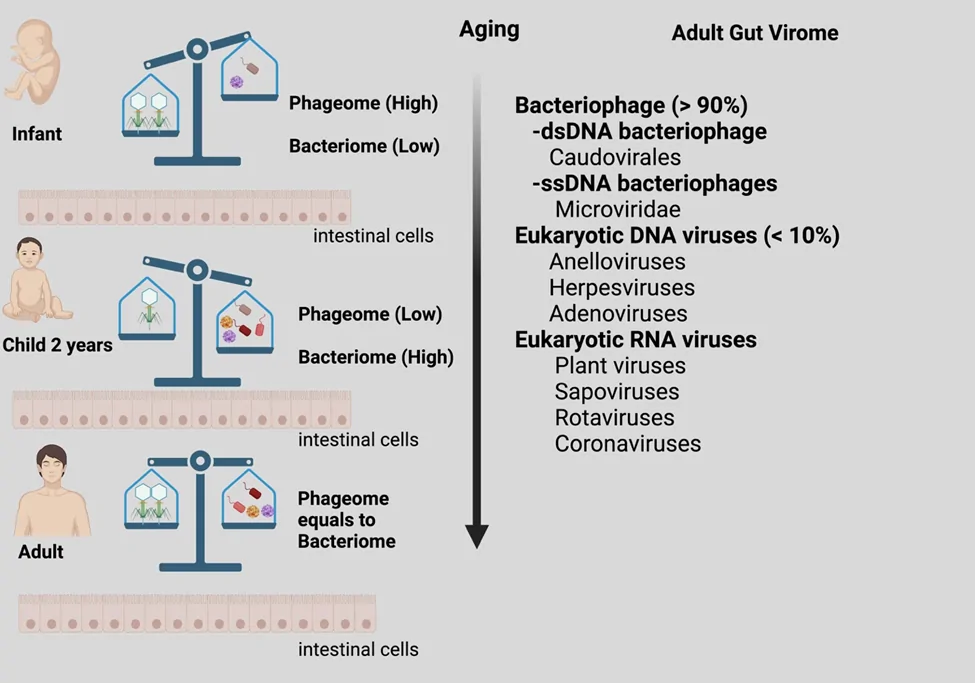
The human gut virome composition changes with the passage of age.

The human virome has a distinct composition across various anatomical regions, such as the blood, GI tract, respiratory tract, urogenital system, and skin. An adult human typically has about 10^^12^ virus-like particles (VLPs) in their gut, which is comparable in number to gut bacteria and correlates with body size [22]. The gastrointestinal system contains the most viruses in the human body [23]. The gut virome of a healthy adult includes DNA viruses (ssDNA and dsDNA) and RNA viruses (ssRNA and dsRNA). Research conducted by [1] and other studies has consistently shown that people have unique gut virome compositions that differ based on location, lifestyle, nutrition, and age [22, 24]. Human gut virome consists of both eukaryotic and prokaryotic viruses that infect bacterial cells. Eukaryotic viruses, such as herpesviruses, anelloviruses, and adenoviruses, account for less than 10% of the virome [23]. Eukaryotic DNA viruses, like herpesviruses, anelloviruses, and adenoviruses, often remain latent and quiescent, modulating host immunity [25]. Eukaryotic RNA viruses are rare in healthy individuals; most intestinal eukaryotic RNA viruses are identified as plant viruses [26]. Pathogenic RNA viruses may be present in the stomach of a human host under stress from infection [24], posing significant health risks through transmission via the fecal-oral route, contaminated food or drink, and person-to-person contact. The RNA virome in the human gut is less studied than the DNA virome because RNA viruses are less stable in samples than DNA viruses, making detection via metagenomic sequencing [27]. In the human gut, phages, also called bacteriophages and bacterial viruses, comprise over 90% of the gut virome, though RNA phages are less common compared to eukaryotic viruses (Fig. 1) [22].

According to some research, the first phages in a baby's gut originate as prophages from the initial bacteria to colonize the area [28], such as *Firmicutes*, *Bacteroidetes*, *Actinobacteria*, and *Proteobacteria*, all acquired during vaginal delivery [29]. For example, it has been shown that breastfeeding transfers prophages carrying *Bifidobacteria* from mother to infant. It was discovered that bifidophages belong to the Caudovirales family, which dominates the early baby phageome and contains very few crAssphages. Between one and three months after *Bacteroides* invade the baby's intestines and start to multiply, crAssphages from the baby gut and mother gut share up to 99% of their genomes, comparable to the colonization of Bifidobacterium and bifidophages [30]. The gut virome of breast milk showed a significant number of phages infecting *Lactobacillus* and *Bifidobacterium,* with very little evidence of eukaryotic viruses [31]. A study was conducted to examine the impact of formula milk and breast milk on the gut virome in infants from America, Africa, and Botswana. The results showed that while breast-fed infants in the American and African groups had few Enteroviruses in their stool, those in Botswana did [31]. Children in resource-poor countries have a wider variety of enteric viral infections compared to children in resource-rich regions, according to other research [32]. These studies suggest that the ecology of the gut virome primarily depends on diet and region. Probiotics are the early bacteria that colonize the gut and aid in shaping the gut microbiome ecology [33].

### 2.2. Animal virome

Recent research has shown that a greater variety of RNA phages are more frequently found in various environmental habitats, such as animal feces, than previously believed [34]. Most intestinal phages found in the human gut are encoded with DNA. The dominant taxa in the gut virome are Caudovirales (dsDNA viruses) at the order level and Microviridae (ssDNA viruses) at the family level [35]. crAssphage is a common phage family known as Intestiviridae within the (newly established crAssvirales order by ICTV in 2021). Different forms of crAssphage have been observed in human feces worldwide, making up approximately 22% to 90% of the relative abundance within the human gut virome [36]. Their connections to human health and disease remain uncertain [37]. Research conducted over 2.5 years on the gut virome of an adult revealed that over 80% of the detected viral contigs remained consistent throughout the study [38]. A year-long longitudinal study of fecal VLPs in 10 healthy adults showed a consistent, individual-specific gut virome composition, mainly consisting of crAss-like and Microviridae bacteriophages. The composition of the human gut virome changes with aging [22].

Studying the viral causes of gastrointestinal disorders in farmed horses is essential because each animal holds significant value. Analysis of dsDNA viruses in horse feces was conducted using metagenomic sequencing with a sequence-independent cloning technique [39]. This method produced many phage sequences from different families, such as *Siphoviridae, Myoviridae, Podoviridae*, and included a clone from a vertebrate Orthopoxvirus. Five eukaryotic enteric viruses were identified in healthy feline fecal samples from Portugal through high-throughput metagenomic sequencing using Illumina MiSeq and BLAST. The feline virome sequences included Picornavirus, Astrovirus, Bocavirus, Rotavirus, and Picobirnavirus. These viruses were shed by healthy, asymptomatic animals, including a novel picornavirus named "Sakobuvirus," which is distantly related to the human virus genera Salivirus and Kobuvirus [40]. The Illumina MiSeq platform was employed to analyze the feline enteric virome of cats from a single shelter in California. The virome observed in this study showed similarities to the Portugal study, including astroviruses, bocaviruses, and picobirnavirus. However, the California research did not detect picornavirus but did find additional viruses from families like *Coronaviridae*, *Circoviridae*, *Herpesviridae*, *Anelloviridae*, and *Caliciviridae* [41]. Besides cats, dogs are also a potential source for cross-species transmission through close contact with humans. A recent paper analyzing the viral metagenome of domestic dogs has been published [42], showing comparable virome families to those found in studies of viruses in cats from Portugal, America, and Canada [43]. An analysis of the gut microbiome in 45 healthy domestic dogs in China using advanced next-generation sequencing revealed high levels of various DNA and RNA virus families, with *Circoviridae*, *Parvoviridae*, *Herpesviridae*, *Astroviridae*, *Coronaviridae*, and *Picornaviridae* being the most prevalent among 63 identified virus families (31 DNA virus families and 32 RNA virus families). Fecal viral contigs from asymptomatic East African farm pigs, analyzed through high-throughput sequencing and metagenomics, showed a mixture of contigs with no matches and others closely resembling known viruses in the GenBank database. The most common genera found included Astrovirus, Rotavirus, Bocavirus, Circovirus, and Kobuvirus, present in both wildlife and domestic animals [44]. Less common contigs in porcine fecal samples included Picobirnavirus, previously noted as prevalent in domestic animal viromes [40, 41], while others like Sapelovirus, Pasivirus, Posavirus, and Teschovirus[44] were not detected in the wildlife or domestic studies reviewed [37]. Some viruses, such as Teschovirus, are exclusive to pigs, while others, including Astrovirus, Rotavirus, Bocavirus, and Kobuvirus, are known to cause disease in humans [45]. A metagenomics study examined the enteric viromes of pigs in the United States. The analysis revealed the presence of core genera in the variable viral contigs, despite the pigs not being asymptomatic but infected with porcine endemic diarrhea virus (PEDV) [46]. Using the Kraken method, the following genera emerged as the most prominent: Mamastrovirus, Enterovirus, Sapelovirus, Posavirus, Kobuvirus, Sapovirus, Teschovirus, Pasivirus, and Deltacoronavirus. All viruses identified in this study of diarrhea-affected farm pigs belonged to four viral families frequently encountered in wildlife and domestic studies: *Picornaviridae, Coronaviridae, Astroviridae, and Caliciviridae* [47].

The dominant virome families in farm animals are not limited to pigs and have also been identified in recent research involving metagenomic studies of healthy and sick broiler flocks in the poultry sector. This research detected *Parvoviridae, Astroviridae, Picornaviridae, Caliciviridae, Reoviridae, Adenoviridae, Coronaviridae*, and *Smacoviridae* in the broiler flocks [44], with *Adenoviridae* and *Smacoviridae* being the only two families of viruses not widely observed in other wildlife research included in this analysis. Some studies might not have included *Smacoviridae*, as it was a newly classified group introduced by metagenomic techniques and officially recognized by the International Committee on Taxonomy of Viruses (ICTV) in 2018 [41]. Additionally, this research showed the presence of Chaphamaparvovirus in all flocks [44], viruses previously associated with bat viromes [39] and healthy cat viromes [45]. The study on broiler chickens supports earlier findings of a high presence of *Chlamydia parvum* in both sick and healthy animals, such as ducks in Australia [46], mice with kidney disease in Australia and America [47], and dogs with various illnesses in America [48].

## 3. Comparative analysis: Animal vs. Human gut Virome

### 3.1 Similarities in composition:

Research on the gut virome in both animals and humans has shown similarities in its composition, especially regarding certain viral types and their functions. These findings open exciting opportunities for further research in the field.

Developments in biotechnological instruments have enhanced our understanding of the gut microbiota and have shown that, contrary to previous beliefs, the number of viruses in the gut may often equal or exceed that of bacteria [22]. They consist of various viruses, such as DNA and RNA viruses [1] as well as prokaryotic (90%) and eukaryotic (10%) viruses. However, limited research on gut RNA viruses might be due to the viruses' instability in culture [27] along with the fact that the composition of gut viruses varies depending on factors like age, location, lifestyle, and diet [22, 24]. The virome includes all single-stranded DNA and double-stranded DNA viruses present in a healthy adult human stomach. According to [22, 24], RNA viruses can also be classified as double-stranded (dsRNA) or single-stranded (ssRNA).

### 3.2 Key differences and factors influencing these differences:

The main components of the human virome include endogenous retroviruses, viruses that infect eukaryotic cells, and bacteriophages (phages) that infect bacteria [38]. The most common organisms on Earth are bacteriophages, and this is also true in the gut, where bacteriophages are the most numerous residents. Most bacteriophage genomes sequenced from human gut or fecal samples come from the ssDNA family *Microviridae* and the dsDNA families *Siphoviridae, Podoviridae*, and *Myoviridae* (order Caudovirales) [11, 12].

Further research has shown that the number of the gut virome in an adult human is estimated to be comparable to gut bacteria, with approximately 10^^12^ virus-like particles (VLPs) per individual, proportional to body size [22]. Human gut virome topologies are highly personalized and differ across regions, lifestyle, nutrition, and age, according to studies conducted by us and others [22, 24].

Similarly, using metagenomics allows us to see how the gut viromes of animals and humans differ in terms of diversity. Whole RNA sequencing has enabled rapid and accurate virus identification by removing the need for costly and time-consuming methods like microscopy or cell culture [48, 49]. These studies have shown that viruses with single-stranded RNA, in both positive- and negative-sense orientations and covering the full spectrum of genome types, infect animals. Animal viruses are classified into five realms, ten kingdoms, eleven phyla, twenty-six classes, thirty-five orders, and ninety-nine families, according to the International Committee on the Categorization of Viruses’ new classification published in July 2021 [50]. However, even with the expanded species sampling enabled by mNGS (metagenomic next-generation sequencing), viruses associated with humans or human activity groups, such as specific phyla or vertebrate classes, continue to dominate our understanding of the animal virome [51]. Reyes and colleagues studied how nutrition affects the gut virome and found that even among people on similar diets, there was significant variation in gut virome [52]. Minot and others challenged this idea, arguing that diet influences the gut virome. They observed that when patients consumed the same food, their gut viromes became more similar but still differed in specific composition (Minot et al., 2011). In the mucosal and luminal viromes of obese mice fed a high-fat, high-sucrose "Western" diet, Kim and colleagues found that temperate phages of the Caudovirales order were notably abundant [53].

### 3.4 Implication of cross-species transmission of viruses

Besides fully adapted human viruses, many animal viruses ingested through contaminated food are also found in the gut virome and have no clear connection to the human virome. HEV causes acute hepatitis; genotypes 1 and 2 are exclusive to humans, but types 3 and 4 can also infect pigs. While genotypes 1 and 2 are common in the human virome and may be adapting to humans, genotypes 3 and 4 are known to infect humans from the animal reservoir [52]. Humans are often infected with HEV, a zoonotic virus; in pigs, however, the infection often goes unnoticed. This proposes that the composition of the virome and clinical features are greatly influenced by the host’s immune status [53].

Additional studies showed that the only known member of the Circoviridae family is the chicken anemia virus [54]. NGS initially identified the first human gyrovirus (HGyV) on the skin surfaces [55]. AGV2, a very similar virus, was found in chickens a few months later. Later, a third gyrovirus, which includes CAV and HGyV/AGV2, was discovered in human stool [56]. The discovery of GyV4, a fourth gyrovirus, occurred in poultry and human stool in China [57]. Some reports suggest that these four gyroviruses are highly common in human feces, possibly due to gyroviruses replicating in humans through cross-species transmission. Similar to plant viruses, passive animal viruses spread through food consumption [58]. Like gyroviruses, cycloviruses, a new genus in the Circoviridae family, are found in the feces of humans and other animals [59].

## 4. Evolution of Gut Virome Diversity

Viruses, especially bacteriophages, play an important role in the mortality and evolution of microorganisms by enabling horizontal genetic transmission (HGT) [60, 61]. This viral activity is believed to significantly influence nutrient cycling and carbon transport in biogeochemical and ecological processes [62], emphasizing their vital role in these functions. Research on the ecological effects of viruses has mainly focused on aquatic environments, especially in marine ecosystems, despite their widespread presence in all ecosystems [63].

The human gastrointestinal tract hosts a wide variety of unidentified microorganisms, including bacteria, archaea, microbial eukarya, and viruses, which have adapted in response to the immune system's selective pressures [64]. Changes in gut microbiota maintenance and composition are strongly linked to human physiology, nutrition, and disease development [65]. An increasing number of studies are connecting changes in gut microbiota to the onset of metabolic syndromes and inflammations such as obesity, diabetes, and inflammatory bowel disease [66, 67].

Considering the significant impact of viruses on host mortality and genetic diversity in the environment, their ecological effect on the gut's microbial communities may also be substantial. The functional redundancy of the gut microbiota not only ensures stability for their survival [64] but also complements metabolic functions not encoded by human DNA [68]. The high frequency of horizontal gene transfer (HGT) among gut bacteria [69, 70] and the abundance of phage-related genes in the human gut microbial metagenome suggest that viruses contribute to maintaining gut homeostasis [68]. Human gut viruses are not well understood and have yet to be recognized as a major component of the gut microbiome. Few studies have investigated the diversity of viruses in the human gut, often focusing on a small number of individuals and employing shotgun library creation with limited resolution. Advances in molecular techniques have greatly enhanced our understanding of viral diversity. There is increasing interest in studying viruses in various environments, driven by the greater diversity and innovations associated with viruses than previously recognized. Single-stranded DNA (ssDNA) viruses, especially those related to macrophages, have been found in abundance and diversity through multiple displacement amplification (MDA) with phi29 polymerase. This method enables the study of ssDNA viruses by using random priming and the selective amplification of circular genomes [71].

Enteric virome diversity varies for each individual, as demonstrated by research on monozygotic and dizygotic twins [72], and correlates with the overall diversity of the gut microbiota. Most of the virome consists of phages and their replication dynamics are closely tied to bacterial populations. This diversity of the enteric virome reflects the complexity and richness of the human gut microbiota, which continues to fascinate and challenge researchers in the field [22, 38].

An analysis of the human virome worldwide identified several viral enterotypes [73, 74]. These enterotypes are distinct clusters of viruses categorized into three main groups based on their primary bacterial hosts (Bacteroides, Prevotella, and Ruminococcus) [75]. The hypothesis must be tested using bioinformatics to uncover links between viral enterotypes and their influence on human health. The intestinal virome undergoes significant changes during the first 24 months after birth [22, 38, 76, 77]. Over time, the enteric virome develops a unique composition for each individual and generally remains stable with minimal variation. Reports suggest that up to 80% of the sequences remain unchanged [22, 72]. Maintaining the stability of the gut virome over time is essential for good health. The integrity of the gut microbiota has been associated with improved psychological well-being [78-80].

Mammals have a diverse range of bacteria in their lower gastrointestinal system, which also hosts a varied population of phages. The virome in fecal samples from mammals and birds was examined using viral metagenomics, uncovering many distinct *Microviridae* sequences. The study focuses on the quantity of bacterial viruses in different animal species, their genome structure, and sequence similarity based on the VP1 protein, a fundamental phylogenetic marker used for classifying Microviridae [81], along with the phylogenetic diversity analyzed in this research.

The virome plays a role in various homeostatic processes and in regulating microbial populations by interacting with phages and bacteria [82, 83]. Stability is disrupted when the gut's normal function is interrupted, leading to dysbiosis, a condition characterized by an imbalance in the gut microbiota [84]. The greater diversity and abundance of viruses in the gut may contribute to diseases such as inflammatory bowel disease [85, 86]. Other health conditions, such as infection with *C. difficile*, type 1 diabetes, cancer, or AIDS, significantly alter the virome [87, 88]. Changes in the gut microbiota have been linked to the development of several illnesses and metabolic disorders [89]. Compared to healthy individuals, patients with ulcerative colitis (UC) showed an increase in mucosa phages and a decrease in the diversity and abundance of phages from the Caudovirales group. Additionally, there was a rise in Escherichia phage and Enterobacteria phage populations in the intestines of UC patients [90]. De Jonge et al. conducted research examining the relationship between metabolic syndrome (MetS) and changes in the gut phageome [86].

The composition and function of the human gut virome undergo a profound and specific evolution across the lifespan, transitioning from a relatively unstable state in infancy to a more stable and diverse adult profile, before shifting toward a distinct and often dysbiotic state in older age [91]. This geriatric virome is frequently characterized by an expansion of viral richness (alpha diversity) and a pronounced shift in community composition (beta diversity) compared to younger adults; however, its most critical functional evolution is a predicted increase in the abundance and activity of temperate phages [18, 92]. This shift towards lysogeny is theorized to be a key adaptation to the aging gut environment, potentially driven by a combination of factors: a declining mucosal barrier allowing for easier phage-bacteria contact, a nutrient-poor milieu from dietary changes that favors the metabolic efficiency of lysogeny, and most importantly, a weakened immune surveillance that reduces the cost of maintaining a prophage [19]. This last point is critical, as the age-related remodelling of the immune system immunosenescence, may directly permit the proliferation of temperate phages, which in turn actively exacerbate immune decline. The functional consequences of this are twofold. First, through phage-driven microbial destabilization, the expansion of temperate phages can mediate the horizontal transfer of antibiotic resistance and virulence factors, while also eroding the diversity and resilience of the bacterial microbiome, depleting keystone taxa responsible for producing immunomodulatory metabolites, such as butyrate [93]. Second, the aging virome contributes directly to immune activation and inflammation. The increased viral particle load provides a chronic source of pathogen-associated molecular patterns (PAMPs), such as viral nucleic acids and capsid proteins, which can persistently engage pattern recognition receptors (e.g., TLR7/8, TLR9) on innate immune cells and the intestinal epithelium [82]. This constant, low-grade stimulation is a plausible mechanistic contributor to the state of inflammation, a hallmark of immunological aging characterized by elevated pro-inflammatory cytokines. Therefore, the aging virome is not a passive bystander but an active ecosystem that co-evolves with and potentially accelerates host immunological decline, creating a vicious cycle of microbial instability and chronic inflammation.

## 5. Gut virome interaction with gut bacteria

**Figure 2.**
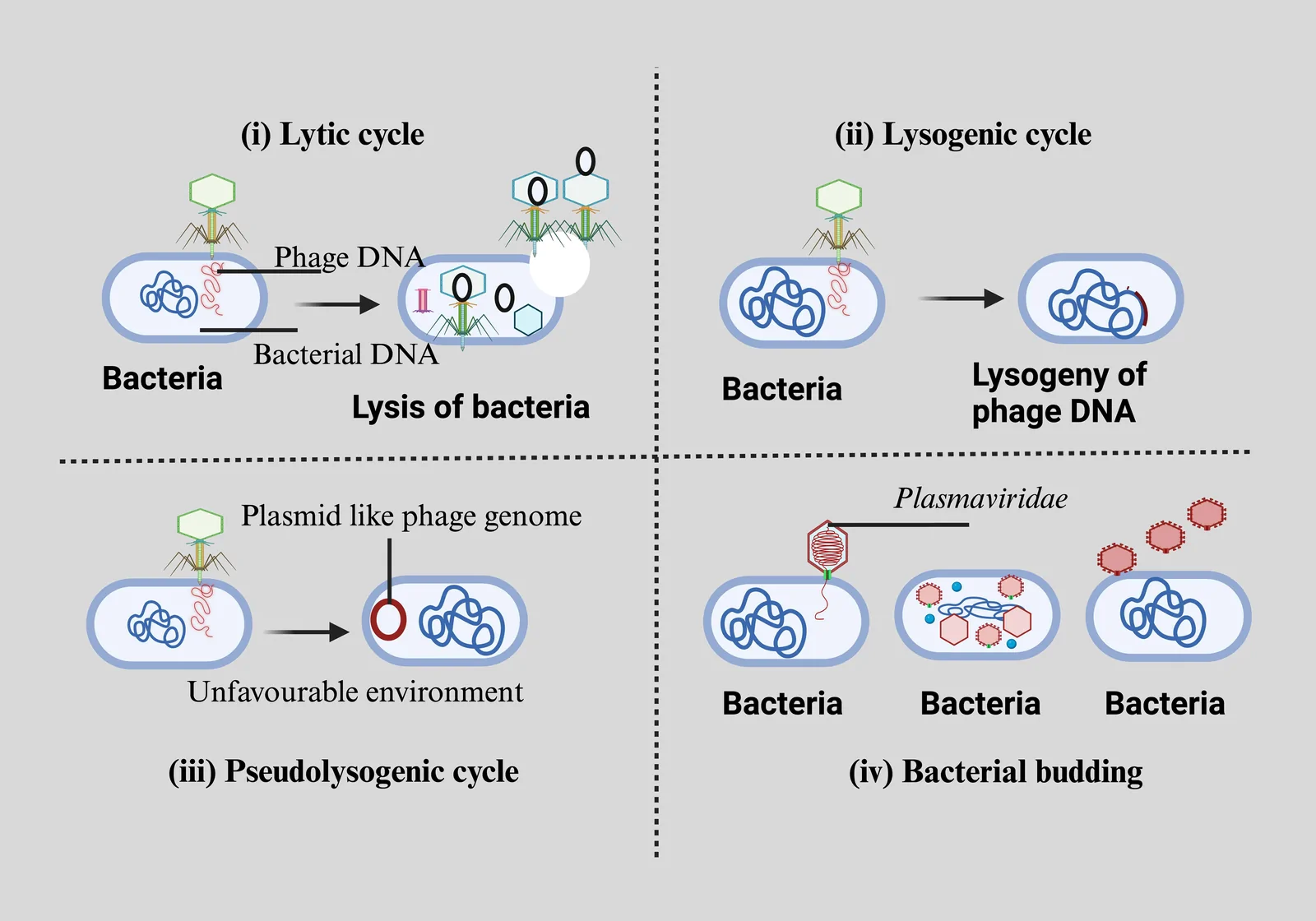
Schematic presentation of phages Life Cycles.

The microbiome consists of infectious entities in the human body that interact with each other and the host. This interaction influences health and disease [94]. Phages are commonly found on mucosal surfaces, in the intestinal lumen, and in feces, where they interact with co-resident bacteria. These phages alter the composition of bacterial communities in the gut through their replication cycles [23]. There are four types of phage replication cycles: bacterial budding, lysogenic/temperate, pseudo-lysogenic, and lytic cycle (see Fig. 2). The two main forms are lysogenic and lytic cycles. The lytic phage (virulent phage) injects its genome into the host bacteria, leading to the production of viral macromolecules and particles (viral offspring), followed by bacterial cell lysis and release of viral progeny. The virulent phage phenotype is common during gut inflammation and environmental stress. Lysogenic phages integrate their genetic material into the bacterial chromosome, altering bacterial phenotypes by horizontal gene transfer, which can include toxin production and antibiotic resistance genes[95]. The phage genetic material can be excised from the bacterial chromosome in response to factors such as diet, chemical inducers, pH or temperature changes, ultraviolet radiation, antibiotics, and inflammatory or oxidative stresses, enabling the switch to a lytic cycle [96-99]. Phages can enter a pseudo-lysogenic state under unfavorable environmental conditions, where the phage genetic material resides in the bacterial cell without replicating or integrating as an episome that resembles a plasmid [100]. The bacterial host may be protected from lysis or death by certain phages, including *Plasmaviridae.* These phages have a bacterial budding life cycle, in which they form buds that emanate from the bacterial cells [101]. Phage-driven intestinal dysbiosis has been suggested by several speculative hypotheses [102].

According to the model of “Kill the Winner,” phages reduce the number of dominant commensal bacteria in the gut. The model relies on a relatively high diversity of bacterial strains within the community. There is little evidence that this happens in the gut; this strategy is only relevant to dominant members of an ecosystem [103]. According to the "Biological Weapon" paradigm, commensal microbes' phages act as weapons against competing bacteria, leading to dysbiosis. Phages benefit their host by killing rival bacteria [104].

According to the "Community Shuffling" paradigm, prophage induction in bacteria can be triggered by biotic conditions such as oxidative stress, antibiotic therapy, and inflammation. This mechanism disrupts the interactions between symbionts and pathobionts and results in lytic infection in bacteria [105]. Besides the three models previously mentioned that rely on the ability of phages to lyse their hosts, phages can also alter the phenotypic traits of bacteria through gene transfer. This process is called the “Emerging New Bacterial Strain” model, where instead of lysing the host cell, phages effectively insert the lysogen into their host [17]. Additional evidence supports this theory, showing that bacteriophage transcription factors enable them to regulate bacterial functions during lysogeny. For example, the enterohaemorrhagic *E. coli* T3SS is activated via the Cro transcription factor of phage, thereby increasing bacterial virulence [106].

Phages influence the functions of the gut bacteriome by regulating host viability and metabolism. Increased resistance to acid was observed in *E. coli* infected with the bacteriophage Φ24_B_ [107]. The phages modulate bacterial gene expression and enhance the virulence and environmental adaptation of host bacteria. They increase the production of exogenous toxins and alter the biofilm structure and metabolism of host bacteria [108]. Examples of clinical bacterial pathogens that acquire ARG through phage transduction include *Staphylococcus aureus* [109], *Clostridium difficile* [110], and *Enterococcus faecalis* [111]. Notably, several subsequent studies have shown conflicting results, suggesting that phageomes associated with humans and animals rarely carry ARGs [112, 113]. Phage-encoded factors can influence gut bacteria's pathogenicity by promoting adherence, colonization, invasion, and the production and transport of toxins (Fig. 4) [114]. The class of enzymes known as adenosine-diphosphate-ribosyltransferases (ADPRTs), encoded by specific phages, has been shown to enhance energy utilization, colonization, and adhesion of gut microbes [115, 116].

**Figure 3.**
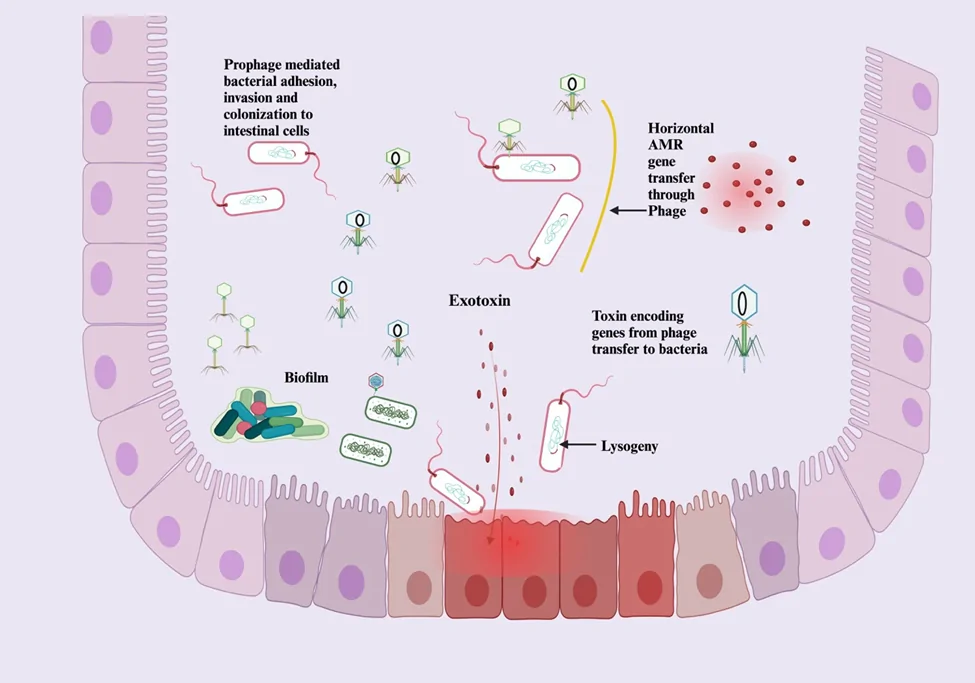
The phenotypic alterations of host bacteria caused by phage infection.

### 5.1 Gut Virome Interaction with Immunity

Viruses and phages are abundant on the gut mucosal surface, where they regulate the host’s immunity [114]. It has been reported that mucin glycoprotein on the gut's mucosal surface binds with Ig-like proteins on the viral capsid. Bacterial invasion of human mucosal and epithelial cells is significantly reduced by weakly adhering T4 phage expressing Hoc proteins on the mucosal surface, which causes aberrant slow diffusion behavior [117]. Every day, approximately 31 billion phage particles pass through human gastrointestinal epithelial cells and enter the bloodstream [118].

The spleen and liver mononuclear phagocyte systems capture and filter phages during circulation, and phage-specific antibodies neutralize them within 24 hours [119]. Phage activity influences the human immune system by promoting bacterial opsonization and identification, regulating cytokine release, and modulating T and B cell activity (Fig. 5) [120]. The expression of type I (IFN-I) and type III (IFN-III) interferons is stimulated via cytosolic sensors such as retinoic acid-inducible gene-I (RIG-I), endosomal toll-like receptors (TLRs) signaling through TRIF and MYD88, and melanoma differentiation-associated protein 5 (MDA5) signaling via mitochondrial antiviral signaling (MAVS) protein [121]. MDA5 and RIG-I play critical roles in antiviral responses against rotavirus, a dsRNA virus that affects the epithelium of the small intestine and causes diarrhea in children [122]. RIG-I triggers the production of interleukin-15 (IL-15), which helps maintain the homeostasis of intraepithelial lymphocytes (IELs) (Fig. 6) [123].

**Figure 4.**
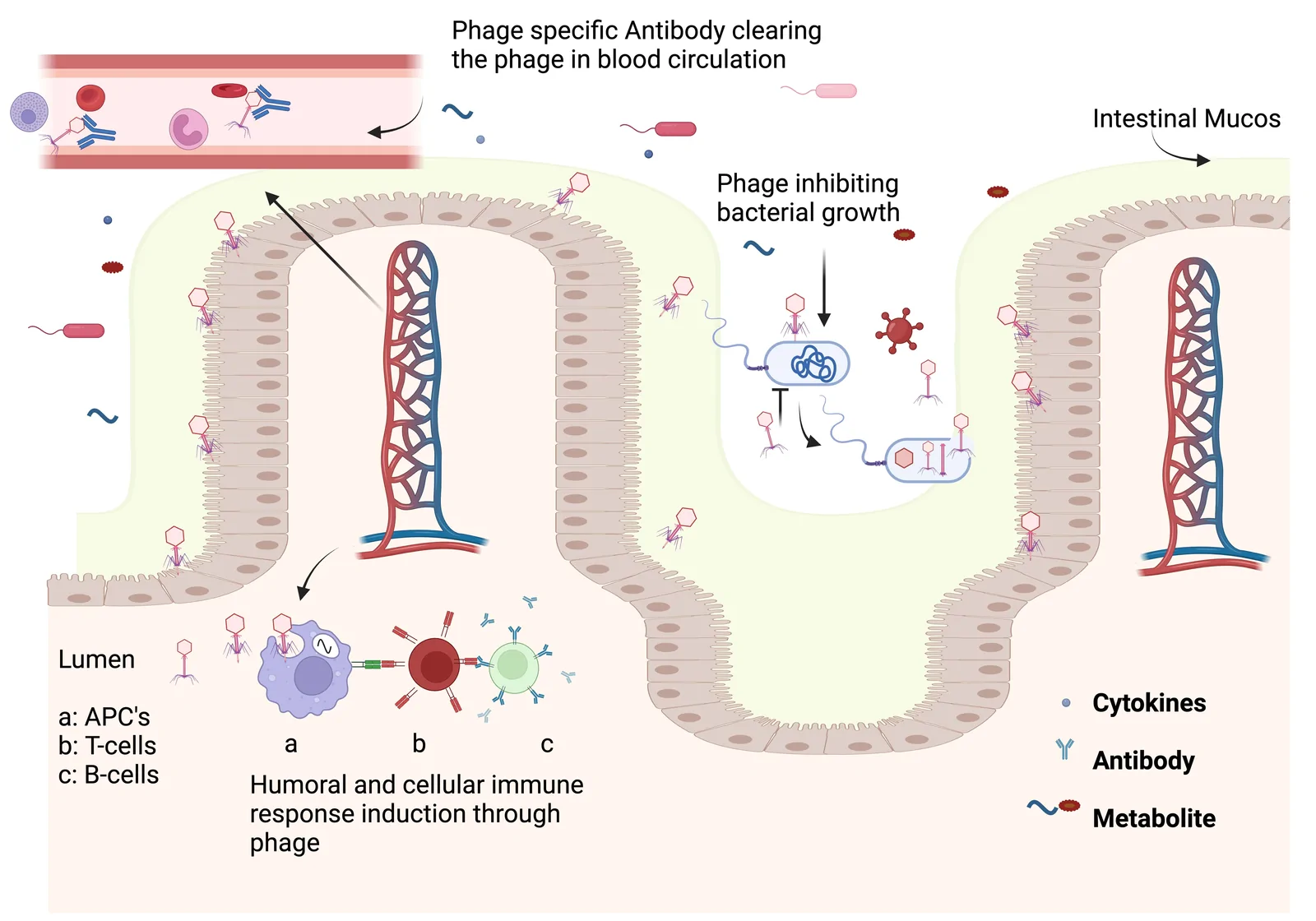
The role of eukaryotic viruses in maintaining the host immunity and human gut homeostasis.

The ssRNA noroviruses also cause human gastroenteritis, and this virus can be detected by myeloid cells via MDA5, TLR7, and TLR3 [124]. Germ-free mice colonized with Murine norovirus (MNV) triggered an immune response that protected against *Citrobacter rodentium* infection and promoted intestinal epithelial cell growth [125]. Immunocompromised mice supplemented with Murine astrovirus (MAV) were protected against enteric infections through the production of IFN-γ. Fecal transplantation and cohousing are used to transfer this protection [126]. It was observed that intestinal viruses might increase colonization resistance in patients with *Clostridium difficile* infection after fecal microbiome transplantation. Remarkably, when it comes to treating patients, filtrated feces are just as effective as unfiltered feces [127]. Bacteriophage abundance and richness were reduced in patients infected with *C. difficile* compared to healthy controls, suggesting that bacteriophages could be the active component of fecal microbiome transplantation (FMT). The successful transplantation was associated with the transfer of Caudovirales species [128]. RNA helicase DHX15 acts as an NLRP6 co-factor to signal via MAVS. Mice with NLRP6 deficiency exhibit blunted IFN-I/III responses, making them more susceptible to oral infection by Murine norovirus and encephalomyocarditis virus [129].

**Figure 5.**
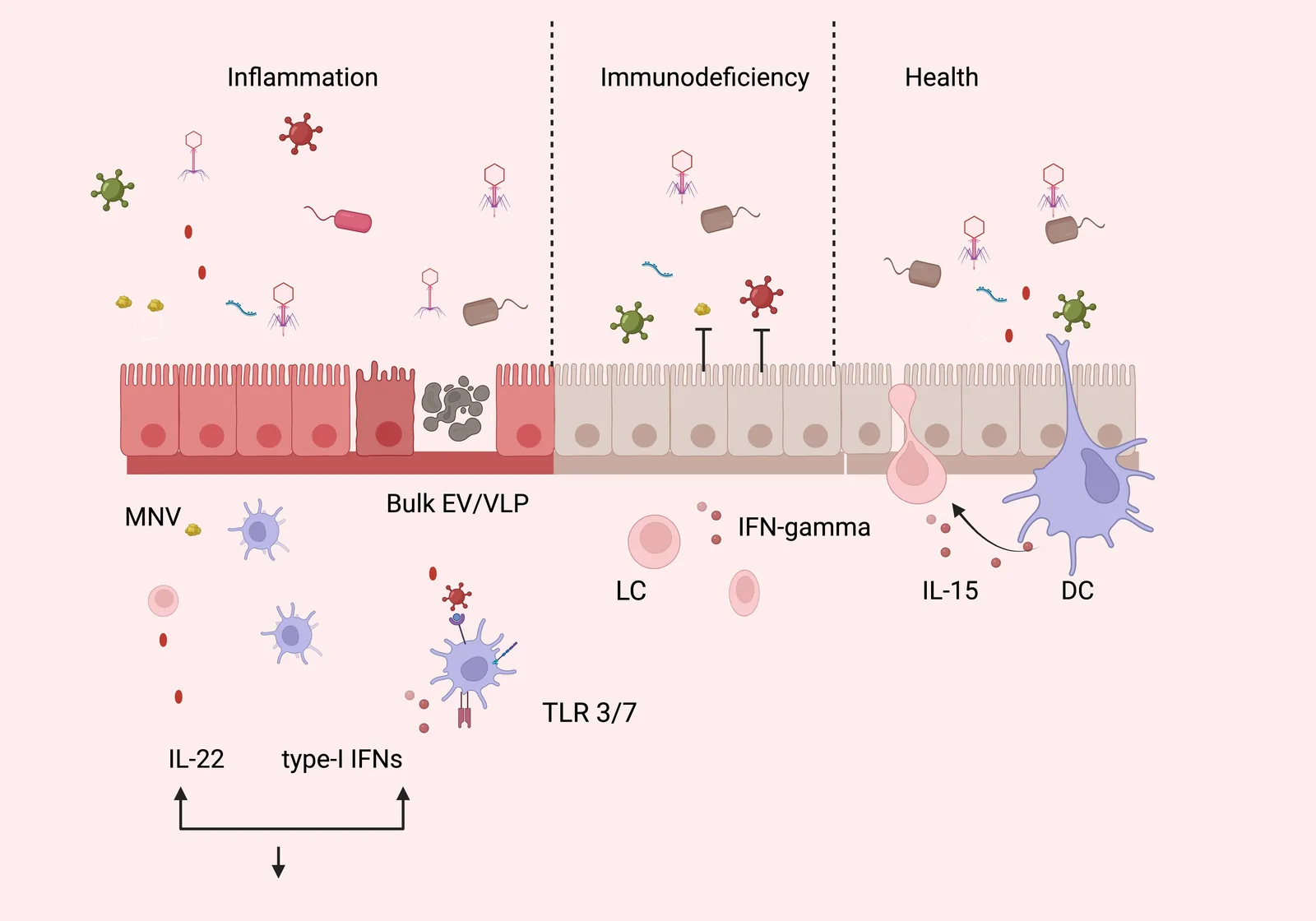
The interaction between the immune system and phages.

According to a recent study, the proliferation of interferon-gamma (IFN-γ) increases in germ-free (GF) mice after feeding with *E. coli* or T4 phages. The CD4+ and CD8+ T cells in the intestinal mucosa produce interferon-gamma (IFN-γ). The *E. coli* phages activate Toll-like receptor 9 (TLR9) and IFN-γ-dependent pathways in a dextran sodium sulfate (DSS)-induced inflammatory bowel disease mouse model, which exacerbates colitis in the mouse [82]. IFN-I (IFNα and IFNβ) binds the interferon-α/β receptor (a complex of IFNAR1 and IFNAR2), and IFN-III (IFNλ) binds the interferon lambda receptor (a complex of IL10R2 and IFNLR1) to initiate antiviral gene expression. IFNAR1 is expressed on many cell types and prevents the dissemination of rotavirus, reovirus, and murine norovirus in the body [130]. The group 3 innate lymphoid cells produce IFNλ and IL-22, which synergistically control intestinal *rotavirus* [131]. The adaptive immune response is enhanced by interferons, which help control viral infections [132]. One potential solution is to boost innate immunity to overcome the gut's insufficient antiviral defenses. Chronic rotavirus infection in mice can be prevented and treated with bacterial flagellin, which does so by inducing the production of IL-18 and IL-22 via NLRC4 and TLR5, respectively [41].

## 6. Gut virome in Animal Health

### 6.1 Contribution to digestion and nutrient absorption

The microbial community of ruminant species has evolved alongside the host, enabling the animal to extract energy from high-fiber, low-quality diets [128]. In addition to providing energy and a variety of short-chain fatty acids (SCFAs) to the host, the diverse microbes and fermentation processes in the rumen of ruminant animals produce carbon dioxide, methane, and hydrogen into the environment. A wide range of organisms, including bacteria, protozoa, fungi, viruses, and archaea, inhabit this anaerobic environment. These microbes depend on each other and are essential for breaking down complex polysaccharides and plant-derived polymers. They also play a key role in exchanging hydrogen, fermentation products, oligomers, and monomers among different species. The intricate relationships between viral phages and their bacterial hosts regulate the behavior and ecological dynamics of several natural systems [130]. Numerous natural systems, such as the marine environment, aquifer sediments, infectious human illnesses, and animal intestinal ecosystems, exhibit viral influences on microbial and cyanobacterial metabolism [131-133]. Using various techniques, including energy generation, cell lysis, replication, and alteration of microbial metabolism by Auxiliary Metabolic Genes (AMGs), viruses impact the ruminal and intestinal microbial communities of cattle [134, 135].

### 6.2 Impact on the Immune System

Hosts and their gut microbiota viruses coexist, leading to low-level immune responses without causing symptoms (host tolerance), all within a dynamic equilibrium [102]. The viral genome and different lysis products produced by bacteria are examples of PAMPs or pathogen-associated molecular patterns. These molecules stimulate the inflammatory response by inducing low-level immune reactions, which may influence the host's resistance to future infections and disease-to-disease. Phages can also trigger innate antiviral immune responses. Activation of toll-like receptors (TLRs), which serve as sensors of innate antiviral immunity, initiates signaling cascades that provoke an inflammatory response. Human infants exposed to bacteriophage particles can produce antibodies, and endotoxin-free bacteriophage particles have been shown to stimulate the production of TNF-α and interleukin-1β (IL-1β) by macrophages [133].

Additionally, the phages in the gut play a crucial role in developing the human immune system to fight infections and maintain healthy gut microbiota. It is known that on the mucosal surfaces of all animal intestines, phage capsids with immunoglobulin-like domains bind to mucin to provide antibacterial protection against infections [134]. Recent studies show that phages can bind to methicillin-resistant *S. aureus* (MRSA) bacteria in the stomachs of host mice to reduce the cytotoxic damage caused by MRSA and boost the immune system's macrophages in mice [135]. Several illnesses and metabolic problems have been linked to variations or abnormalities in the ecology of the gut microbiota [136].

However, immunodeficiency can also influence the composition of the gut virome. According to [137], immunodeficiency caused by lentivirus infection is linked to a significant increase in the intestinal virome. This is observed with pathogenic simian immunodeficiency virus (SIV). Research by the same author suggests that the composition of the intestinal virome may both contribute to AIDS development and serve as a predictive marker of HIV progression [137].

Parallel to this, viromes can modulate their host's immune system by recognizing the host's innate immune sensors and triggering the release of interferons and other cytokines. This initial response encourages the development of more antiviral genes, antibody-mediated neutralization, and enhances the ability of NK cells and CTLs to destroy virally infected cells [138]. Conventional antiviral effectors are associated with the release of immunosuppressive cytokines, including interleukin-10 (IL-10), regulatory T (Treg) cell activity, and increased expression of inhibitory receptors on effector T cells. These mechanisms work to prevent an uncontrolled antiviral immune response from causing excessive harm to the host [139].

### 6.3 Examples of animal studies highlighting the importance

The importance of the gut virome for animal health highlights its role in shaping the gut microbiota, aiding digestion, modulating immune responses, and reacting to environmental factors. As research advances, it offers insights that can help develop strategies to improve animal health and optimize livestock production [140, 141]. Additionally, its significant influence on the phagosome and gut microbiota has been shown to be crucial for maintaining and regulating host health. For example, increased verbal memory and executive function in the brain have been linked to higher concentrations of Caudovirales and Siphoviridae in the gut microbiomes of both humans and animals [142]. Moreover, the phagosome in the gut may enhance the human immune system to reduce infections and maintain a healthy gut microbiome [143]. Studies have shown that bacteriophages with capsids containing immunoglobulin-like domains can adhere to the mucin on the gastric mucosal surfaces of all animals, offering antimicrobial protection against infections [134].

Another study indicated that when phages attach to bacteria in the stomachs of mice, they stimulate macrophages in the immune system, reducing the cytotoxic damage caused by methicillin-resistant *S. aureus* (MRSA) [135]. Several diseases and metabolic issues are linked to changes or disruptions in the gut microbiota`s ecology. For example, people with ulcerative colitis (UC) have higher populations of phages in their mucosa and lower Caudovirales phage abundance and diversity in their stomachs compared to healthy controls. Additionally, UC patients tend to have increased gut phage populations of Enterobacteria and Escherichia [24, 89].

## 7. Role of gut virome in human health

### 7.1 Beneficial Impact

Viruses can act as mutualistic symbionts that provide benefits to their host rather than being purely harmful parasites. They can also be advantageous to human health. For example, the production of IFN-γ during latent *herpesvirus* infection increases resistance to *Yersinia pestis*, *Listeria monocytogenes*, and *Influenza-A* in the host [144]. Intranasal inoculation of Murid herpesvirus *4* (MuHV-4) in mice, which establishes latency, shows that latency increases the number of natural killer (NK) cells containing GzmB protein. GzmB kills tumor cells through granulation, and natural killer cells boost IFN-γ production. IFN-γ plays a key role in regulating the adaptive immune response and defending against bacteria, viruses, and tumors [145]. Higher levels of *Siphoviridae* and *Caudovirales* in the gut have been linked to better verbal memory and cognitive control in the brain [142]. The gut virome supports healthy microbiota and enhances the host immune system. Phages attach to bacteria on the gut lining in mice, reducing the cytotoxic effects of methicillin-resistant *Staphylococcus aureus* (MRSA) [135]. Murine norovirus (MNV) protects mice treated with antibiotics from lung damage caused by *Pseudomonas aeruginosa* [146] and from DSS-induced intestinal infection [147]. MNV also promotes colonization resistance in mice treated with ampicillin [124]. Phages from the *Picovirinae* subfamily, targeting cytolytic *E. faecalis* in the mammalian gut, help reduce alcohol-induced liver damage and cytolysin production [49]. γHV-68 alters lung macrophage composition and provides protection against allergic asthma [148].

## 8. Gut virome and diseases

### 8.1 Link to diseases in animals:

The link between the phagenome and disease is further supported by animal studies. For example, in rodent colitis, gut phage communities have been shown to change, with the most notable change being a decrease in the excess phages of Clostridiales [149]. Additionally, the gut phagenome may be connected to viral infections from external sources. One study examined changes in the intestinal virome in mice infected with Coxsackievirus B3. The results indicated that infection with the foreign B3 virus significantly increased levels of Caudovirales and significantly decreased the number of Microviridae in the gut virome. Therefore, these findings support the idea that different diseases are linked to the gut phagosome [150]. Similarly, the gastrointestinal tract is chronically inflamed in inflammatory bowel disease (IBD) [90]. GI inflammation is largely classified into two groups based on the affected anatomical areas: ulcerative colitis (UC), which usually appears clinically as large bowel inflammation, and Crohn's disease [151], which can influence any region of the GI tract. A study examining changes in the intestinal virome of mice infected with Coxsackievirus B3 found that the B3 virus infection led to a notable rise in Caudovirales and a considerable drop in Microviridae in the gut virome. These results confirm the connection between the gut phageome and the emergence of different diseases [152]. There is still much to learn about the etiology of inflammatory bowel disease (IBD). Still, several animal studies have demonstrated that gut microbiomes are a causal factor in the disease. This is supported by the disruption of normal gut microbiota [85].

## 9. Negative impact on human health

The mounting evidence shows that gut virome regulates intestinal and extra-intestinal disease pathogenesis.

### 9.1 Inflammatory bowel disease (IBD)

Ulcerative colitis (UC) and Crohn's disease are two chronic forms of IBD [86]. Crohn's disease [151] affects the GI tract, while ulcerative colitis (UC) primarily targets the colon. Both conditions cause inflammation in the gut and disrupt the digestive system [153]. IBD significantly impacts patients' quality of life. The composition of the gut virome may influence disease progression, although the exact mechanisms remain unknown [154]. In Crohn's disease, there is a notable increase in *Escherichia*, *Faecali* [155], *Caudovirales [156]* crAssphage, *Lactobacillus*, *Enterococcus*, and *Lactococcus* [157] bacteriophages. In ulcerative colitis, Caudovirales and *Enterobacteria* phages are more abundant [86]. The *Podoviridae, Myoviridae*, and *Siphoviridae* morphotypes are ten times more concentrated in UC patients [158]. IBD may be linked to a shift in bacteriophage populations from virulent to temperate phages [156]. No diversity was observed, and expansion of *Microviridae* was found in both CD and UC patients [85].

The abundance and diversity of human endogenous retroviruses were observed in the samples of IBD colon during several studies of endogenous retroviruses and eukaryotic viruses. These studies suggested that HERV expression in the colon correlated with Herpesviridae (Epstein-Barr Virus) infection [159]. The mucosal surface of IBD patients has a higher concentration of EBV and CMV than controls. Inflammatory and non-inflammatory mucosa had similar EBV virus loads, and treatment regimens failed to address them. These results indicate that EBV is responsible for the onset of IBD rather than disease progression [160]. When inflammatory bowel disease was detected early, a specific eukaryotic gut virome signature was identified through metagenomic analysis of the intestinal mucosa. *Hepadnaviridae* transcript levels were significantly higher, while *Tymoviridae* and *Polydnaviridae* transcript levels were lower in the intestinal mucosa of early-diagnosed UC patients compared to controls. Additionally, in the mucosa of CD patients, there was a lower abundance of *Virgaviridae* and a higher abundance of *Hepeviridae* compared to controls [161].

### 9.2 Diarrheal disease

Foodborne diarrheal diseases have various causes [162] but the main agents are eukaryotic RNA viruses [163]. Reports indicate that rotavirus is the leading cause of death, followed by norovirus and adenovirus in diarrheal patients under five years old (27%) [80]. These viruses are rarely seen in healthy individuals but can become dangerous over time during specific conditions like intestinal injuries, weakened immunity, and gut microbiota imbalance. Viruses act as opportunistic pathogens under adverse circumstances [30]. Changes in the gut virome have been observed during diarrhea associated with *C. difficile* and SARS-CoV-2 infections [24, 90]. Low levels of *Microviridae* and high levels of *Anelloviridae* and *Caudovirales mark CDI-related diarrhea*. Significant links have been found between *Proteobacteria, Actinobacteria*, and *Caudovirales* in CDI [128]. Salmonella Typhimurium infection causes diarrhea in a mouse model, and the production of free phages and their subsequent lysogenic conversion increase due to intestinal inflammation. The lysogenic conversion in P2-like phages (SopEΦ) may worsen intestinal inflammation. This inflammation triggers the bacterial SOS response, leading to bacterial lysis, prophage production, and increased dysbiosis [164].

### 9.3 Human immunodeficiency virus

The human immunodeficiency virus causes acquired immunodeficiency syndrome (AIDS). It has been shown that changes in the bacterial microbiome and enteric virome due to HIV infection may influence the progression of AIDS [165]. The gut virome observed during metagenomic studies of HIV-positive individuals reveals that AIDS develops when peripheral CD4 cell levels are low. The gut is a key site for HIV infection, specific immunological responses to HIV, and pathology [166]. Increased vulnerability to secondary infections and other immunopathologies characterizes AIDS. A plasma virome study found that Anellovirus DNA was present in HIV-suppressed participants, and nucleic acids from Pegivirus A (GBV-C), hepatitis B virus (HBV), and hepatitis C virus (HCV) were detected in some HIV-infected patients on antiretroviral therapy (ART). Furthermore, no correlation was observed between Anellovirus genotypes and elevated T-cell activation levels [167]. Anellovirus levels were higher in patients with lower CD4+ T-cell counts. Patients with AIDS and lower CD4+ levels have a higher likelihood of human endogenous retrovirus presence compared to those with higher CD4+ counts. Changes in the plasma concentration of commensal viruses are associated with AIDS progression [168].

The *families Papillomaviridae, Anelloviridae, Genomoviridae, and Herpesviridae* were identified in swab samples from women with cervical co-infections of HIV and HPV. The prevalence of papillomaviruses was higher in women with precancerous cervical lesions. An inverse relationship was observed between the host's CD4+ T-cell count and the amount of Anellovirus. A greater presence of anaerobic bacteria in the vaginal microbiome was linked to higher rates of herpesvirus or genomovirus in women [169]. Semen samples from HIV-positive men were examined in another study. Multiple HPV genotypes, cytomegaloviruses (CMVs), and anelloviruses were detected. Individuals with active HIV viremia shed viruses more often [170].

### 9.4 Graft-Versus-Host Disease

Herpesviruses, anelloviruses, polyomaviruses, and papillomaviruses are among the persistent DNA viruses that are more frequently detected and have more sequences in enteric GVHD patients, according to research that investigated the dynamics of the virome in enteric GVHD and allogeneic hematopoietic stem cell transplantation (HSCT). Phage richness was also found to be lower in these patients. Unexpectedly, picobirnavirus was linked to early post-transplant GVHD. It was prognostic of severe GVHD of the enteric system and was associated with increased fecal levels of α1-antitrypsin and calprotectin, both indicators of GVHD severity [171].

### 9.5 Cancer

It has been investigated that viruses are responsible for 10-15% of all cancer cases. RNA viruses, such as human T-lymphotropic virus-1 and HCV, as well as DNA viruses like simian virus 40, HBV, HPV, EBV, and Merkel cell polyomavirus, have been linked to cancer development [166]. Colorectal cancer (CRC) is prevalent, ranking second in mortality and third in incidence worldwide. Research has shown that gut microbiota plays a crucial role in the development, progression, and treatment of colorectal cancer [167]. Metagenomic analysis of stool samples from CRC patients revealed variations in the intestinal virome's richness and diversity. Viral genera such as Phikzlikevirus, Tunalikevirus, Orthobunyavirus, and 19 others served as markers to distinguish between CRC and non-CRC individuals. Enrichment of Inovirus and Tunalikevirus phages, which infect Fusobacterium, pks-positive genotoxic E. coli, and bft-positive enterotoxigenic Bacteroides fragilis, has been associated with CRC development and demonstrated trans-kingdom microbial interactions [168]. Reports indicate that certain Inovirus species regulate bacterial biofilm formation, aiding in colon cancer development. Additionally, a significant reduction in crAssphages and Enterobacteria phages, along with decreased bacterial diversity particularly of anti-inflammatory and butyrate-producing bacteria—was observed in CRC compared to non-CRC samples [170]. Preliminary data suggest that EBV infection may induce mutagenesis in intestinal cells, potentially contributing to colorectal cancer development [171]. Moreover, studies have documented that microvesicles produced by EBV-infected B cells can transfer EBV-derived molecules to intestinal epithelial cells, possibly leading to oncogenic transformation [172].

Human polyoma virus 2 is involved in CRC carcinogenesis according to a meta-analysis [172]. JCV encodes the transforming gene T-antigen (T-ag), which is involved in inactivating tumor suppressor proteins [173, 174] and deregulating signaling pathways through β-catenin [175], thus inducing malignant transformation and chromosomal instability in colonic cells [176]. Human cytomegalovirus also plays a role in CRC progression [177-180]. CRC cells infected with CMV had higher levels of Wnt signals, which are linked to cell migration and proliferation [181]. CMV-infected CRC cells also contain Toll-like receptor (TLR) 2 pathway components such as TLR4, NF-κB, and TNF-α [182]. Additionally, CMV-infected CRC cells express cyclo-oxygenase-2 and Bcl-2, proteins associated with colon cancer development and progression [183].

### 9.6 Obesity and Diabetes

Diabetes and obesity are two common metabolic diseases worldwide [184]. Type 1 diabetes (T1D) results from a complex interaction between environmental exposures and genetic predisposition. There is increasing evidence that environmental factors, especially bacteria and viruses, contribute to the development of T1D [185]. A review of studies in animal models suggests that changes in gut bacterial composition often occur before disease onset [186]. Studies have shown that T1D patients have higher levels of short-chain fatty acids other than butyrate, which is produced by gut bacteria. Hence, the gut microbiome might directly cause islet destruction [187].

Additionally, insulin deficiency may stem from autoimmune death of β-cells following viral infection, which can activate autoreactive T cells and lead to autoimmune disease [188]. Studies on the gut virome of pregnant women with an d without T1D reveal that pregnant women's guts are frequently infected with eukaryotic viruses. The presence of tobamoviruses and picobarnaviruses is more common in pregnant T1D patients than in pregnant non-diabetic women. Pregnant T1D patients also show higher levels of three EV-B viruses (ECHOvirus E-18, CVB3, and CVB4) compared to non-diabetic pregnant women. Conversely, non-diabetic pregnant women have higher levels of four EV-A viruses (CVA14, CVA5, CVA16, and CVA10) [189]. A recent study found that in children with T1D, changes in the gut virome occur before the development of autoimmunity. The study also noted that fecal samples from T1D children contained greater amounts of Picornaviridae and Circoviridae genetic material than samples from healthy controls [190]. According to research in Asian and African countries, children with and without T1D do not differ significantly in the prevalence of eukaryotic virus species or genera. However, T1D children showed more frequent HERV signals, which warrants further investigation [191].

Obesity-related insulin resistance is the cause of metabolic disease type 2 diabetes (T2D), and several studies have shown links between T2D and the gut microbiota's composition [192]. The metagenomic sequencing analysis of fecal samples from healthy adult controls and T2D patients was evaluated in a case-control study. The T2D group exhibited a significantly higher number of gut phages, and seven phage operational taxonomic units (pOTUs) that were particularly specific to T2D were identified to consist of two Podoviridae, four Siphoviridae, and one unclassified family. The *Pseudomonas, Lactobacillus, Escherichia, Entero-bacteria*, and *Staphylococcus* were the hypothesized bacterial hosts of these pOTUs. It was found that there is a complex core interaction between phages and bacteria in the human gut ecosystem, indicating that co-variation with changing bacterial hosts alone is not sufficient to explain significant changes to the gut phageome [193]. Further research has revealed synergistic changes in the intestinal bacteria and phage in T2D patients, including a higher abundance of bacterial *Enterobacteriaceae* family and Caudovirales family, the predatory phages of *Enterobacteriaceae* [194]

One global health issue that negatively affects quality of life is obesity. Several studies have shown a link between obesity and abnormalities in microbiome composition. One study compared the gut viromes of obese patients before and after therapy and found that the gut virome composition changed during obesity intervention. The gut virome of obese subjects had a lower alpha diversity index before treatment compared to after treatment. Before treatment, only four viruses were detected in the core virome, but after treatment, nearly 13 viruses were detected, increasing the diversity of DNA viruses [195]. A study on serum samples from children and adults in Qatar found that lean individuals had lower seroprevalence and seropositivity for herpes simplex virus type 1 (HSV-1) compared to obese subjects. Obesity was positively correlated with a higher presence of antibodies against HSV-1/2 peptide epitopes, suggesting a potential role for viral peptides in adipogenesis [196]. Bacteriophages mainly belonging to the Caudovirales family were found in the gut dsDNA virome from fecal samples of school-aged children. Patients with obesity and metabolic syndrome tended to have higher Shannon diversity and phage abundance. The presence of multiple phage contigs was linked to anthropometric measures, gut bacterial taxa, and biochemical markers, including high body mass index and elevated glucose and lipid levels in individuals with obesity and metabolic syndrome [197]. The diversity and richness of the gut virome were lower in obese and T2D patients than in lean controls. Eleven viruses, including phages from *Lactobacillus, Geobacillus*, and *Escherichia,* were more common in obese individuals. The broad trans-kingdom interactions between bacteria and viruses observed in lean controls were significantly reduced in those with type 2 diabetes and obesity [184]. An experiment on mice showed that the total viral DNA and RNA in the feces of obese mice was higher than in normal controls; this increase was closely associated with metabolic markers such as fasting blood glucose, fat mass, and body weight. The overall viral load was negatively correlated with Bacteroidetes and Bifidobacterium, and positively correlated with Firmicutes [198]. The *Siphoviridae* family of viruses was significantly decreased in the fecal virome of high-fat diet (HFD)-induced obese mice, while viruses from the families *Mimiviridae, Microviridae*, and *Phycodnaviridae* increased. The study also revealed that lysogenic phages, especially in the *Siphoviridae* family known for lysogenic lifestyles, were reduced in the HFD group [199].

### 9.7 COVID-19

The SARS-CoV-2 is an RNA virus, which is the causative agent of coronavirus 2019 (COVID-19) disease. COVID-19 causes respiratory illness in humans. Enteric involvement was observed in COVID-19 patients, with 5%-33% experiencing gastrointestinal symptoms such as diarrhea, vomiting, and nausea [200]. A mouse model study found that SARS-CoV-2 infection affects the gut microbiome, and the microbiome's composition was linked to disease severity and recovery processes [1]. The immune dysregulation induced by SARS-CoV-2 disrupts the microbial and viral environment in the human body, leading to long-term COVID-19 or post-acute sequelae [201]. A case-control study showed that pepper mild mottle virus (RNA virus), derived from diet, was underrepresented in COVID-19 patients. In non-COVID-19 controls, their feces contained bacteriophages making up most DNA viruses. In contrast, COVID-19 patients had phages of Escherichia and Enterobacter, along with an enrichment of eukaryotic viruses. The host interferon response and gastrointestinal inflammation were associated with phage abundance. The expression of virulence-related genes, inflammation, and stress during SARS-CoV-2 infection indicated that viruses play a key role in the host's immune response. Studies revealed that SARS-CoV-2 remains transcriptionally active in feces even after clearance from the respiratory system. The live virus may persist in the gut, increasing the risk of fecal-oral transmission. Patients with high SARS-CoV-2 viral loads exhibited a higher prevalence of bacterial species such as *Morganella morganii, Streptococcus infantis, Collinsella tanakaei,* and *Collinsella aerofaciens,* along with greater functional capacity for glycolysis, amino acid biosynthesis, and de novo nucleotide biosynthesis compared to patients with low or no viral presence. Additionally, the gut virome of COVID-19 patients included eight bacteriophage lineages and eleven eukaryotic viruses. There was a significant underrepresentation of 16 prokaryotic virus lineages and the plant-derived RNA virus pepper mild mottle virus in COVID-19 patients [202]. The dysbiosis of the gut virome and bacteriome persists long after hospital discharge [24].

### 9.8 Liver diseases

The gut-liver axis enables the liver to communicate with the gut in both directions, making it a crucial organ for host metabolism [203]. The study on alcohol-associated liver disease (ALD) patients reported that ALD altered the composition of the gut virome, which acts as a potential driver of ALD. ALD patients have higher viral diversity, particularly in alcoholic hepatitis (AH). Intestinal phages such as *Enterococcus, Escherichia, Enterobacter,* and *Staphylococcus,* along with eukaryotic viruses like *Herpesviridae* and *Parvoviridae,* increase in the AH group. The mortality and severity of AH are linked to a high proportion of gut phages. A study on non-alcoholic fatty liver disease (NAFLD) found that the diversity of the gut virome is low in NAFLD patients. The severity of NAFLD correlates with a lower proportion of gut phages [204]. Human adenovirus also acts as a risk factor in the progression of NAFLD [205]. Alterations in the gut virome are additionally associated with the progression of liver cirrhosis [206].

## 10. Therapeutic Applications

### 10.1 Fecal Microbiota Transplantation

Fecal microbiota transplantation (FMT) involves transferring enteric phages from a donor to a recipient. The diversity, richness, and composition of phage communities in recipients have been found to resemble those of the donors [207]. A recent study showed that the effectiveness of FMT for CDI patients was linked to the colonization of donor Caudovirales bacteriophages [90, 208]. Additionally, a correlation was observed between the therapeutic success of FMT and the counts of microviridae after treatment in recipients [209]. This type of treatment has also been studied in instances of GVHD, T2D, and irritable bowel syndrome, besides CDI. A meta-analysis and comprehensive review of FMT therapy for active UC reported higher rates of endoscopic and clinical remission, with no significant increase in adverse events compared to placebo controls [210]. Furthermore, FMT has been shown to potentially treat severe colitis associated with GVHD after HSCT. Repeated FMTs led to a consistent increase in diversity within the virome, mycobiome, and gut bacteriome. The abundance of Caudovirales bacteriophages increased, while TTV decreased following FMT [202]. A randomized clinical trial indicated that FMT might enhance the duration and level of microbiota engraftment in obese T2D patients. The combination of FMT and lifestyle changes improved liver stiffness and profiles in these patients [40]. Methods such as prebiotic and probiotic use, FMT, and dietary interventions are among those aimed at modifying gut microbiota to prevent colorectal cancer and improve treatment responses [211]. FMT also shows promise in reducing side effects for CRC and overcoming resistance to immunotherapy. Phage transfer can induce T-cell cross-reactivity with cancer antigens, potentially boosting the immune response to immunotherapy [212]. The regeneration of gut microbiota and a proportional increase in regulatory T cells within the colonic mucosa suggest FMT's effectiveness in treating colitis related to immune checkpoint inhibitors [213]. During and after the COVID-19 pandemic, screening protocols for FMT are essential for both donors and recipients, as the bidirectional gut-lung axis during COVID-19 infection can influence the lungs and gut directly (through gut microbial metabolites and ACE2 receptors) and indirectly (via the immune system) [214].

### 10.2 Faecal Viral Transplantation

FVT, also known as fecal filtrate transplantation or FFT, is a refined version of FMT that removes fecal bacteria and lowers the risk of bacterial diseases associated with FMT. It has been proposed to be used therapeutically for treating gastrointestinal disorders. The study found that FVT promotes the growth of *Akkermansia muciniphila* (AKM) and *Lacticaseibacillus rhamnosus* GG (LGG) probiotic species in the mice gut. Results showed that FVT-treated mice have a higher abundance of probiotics compared to controls [215]. Healthy stool samples are collected from one person, and the healthy gut virus populations are then transplanted into the colon of another person[216]. Further research indicates that, unlike FMT, transferring fecal filtrate, which includes bacteriophages, metabolites, and bacterial components supporting normal intestinal health was sufficient to eliminate symptoms in individuals with CDI and restore normal bowel habits [217]. A pilot study using a mouse model suggested that autochthonous FVT can modify dysbiotic gut microbiota caused by antibiotic treatment. The FVT reduces the side effects of antibiotics [218]. Another study evaluated the effectiveness of FVT compared to FMT in preventing pigs from developing necrotizing enterocolitis. It was found that FVT was more effective than FMT and had no known adverse effects. FVT changes the gut microbiome, including decreases in *Proteobacteria* and increases in the gut virome within the ileal mucosa [219].

### 10.3 Phage Therapy

A new approach called "phage therapy" uses bacteriophages to specifically target and eliminate harmful microbes. This innovative method offers advantages over conventional antibiotics, FMT, and FVT, which are not specific to pathogenic bacteria and may also destroy healthy gut flora. Therefore, phage therapy's ability to selectively remove these harmful germs could represent a significant breakthrough in disease treatment. Due to its specificity and effectiveness, phage-based therapy has the potential to become the standard medical treatment for gastrointestinal disorders. The United States has not yet approved phage therapy for human use. There are very few instances of the US Food and Drug Administration (FDA) approving the single-use Investigational New Drug program for experimental phage [220, 221]. Research on phage therapy is ongoing, with its promise as an antimicrobial treatment being explored worldwide. Animal models have been used to investigate potential clinical applications, which are still in early development stages. Currently, lytic phages form the backbone of phage therapy, often combined into "a phage cocktail" to enhance effectiveness [222-224]. Phage therapy shows promise in treating extra-intestinal disorders in preliminary studies. Phages derived from the cytolytic bacterium *Enterococcus faecalis* that can selectively target intestinal cytolysin-positive *E. faecalis* to prevent its translocation to the liver and thereby reduce AH have been identified [49]. A sewage sample used to isolate *Myoviridae* lytic bacteriophage PDX has shown efficacy against the diarrheal pathogen enteroaggregative *E. coli* (EAEC) in mouse models both in vivo and in vitro, suggesting that PDX could be a useful treatment for travel-related and pediatric diarrhea caused by EAEC [50]. The pathovar *E. coli* known as adherent invasive *E. coli* (AIEC) can cause both short-term and long-term diarrhea, as well as inflammatory bowel disease (IBD) [225]. Phage therapy is an attractive option for treating AIEC infections or colonization in clinical settings due to the challenge of multi-drug resistance [226]. Research demonstrated that a lytic phage cocktail targeting AIEC significantly reduced LF82 levels and improved intestinal inflammation [225]. Another study involving mice with AIEC-associated intestinal tumors found that targeting AIEC with specific phages reduced tumor burden and increased survival by activating the TLR9-dependent immune response and decreasing AIEC colonization [82]. It is believed that inhaled phage therapy could revolutionize the management of bacterial respiratory infections, especially those caused by antibiotic-resistant bacteria [227]. Several investigations have shown that phage therapy holds great promise for treating bacterial infections in COVID-19 patients [228]. Additionally, phages were found to reduce excessive inflammatory responses in COVID-19 patients by inhibiting NF-κB activation and ROS formation [229].

### 10.4 Phage Engineering

New findings in phage-based therapy have prompted investigations into the potential clinical uses of combining phages with genetic engineering. Specifically, phage therapy for CRISPR-Cas9 system delivery to the gut for microbiome genome editing has gained attention [230]. The refinement of phage therapy is advanced by engineering the structure and functions of phages [231, 232]. According to a recent study, the CRISPR-Cas3 system effectively removed genes associated with the lysogeny form of *C. difficile* phages and successfully transformed them into obligately lytic (virulent) phages. In vivo and in vitro studies provided evidence of its ability to specifically target and kill *C. difficile* in mouse models [232]. Another study demonstrated that *Fusobacterium nucleatum* (a tumor-causing bacterium) infection treated with covalently bonded azide-modified phages attached to dextran nanoparticles coated with irinotecan (an anti-tumor drug) significantly enhanced the effectiveness of first-line chemotherapy for colorectal cancer (CRC) without causing serious side effects. Since the engineered phage-guided nanoparticles precisely targeted the tumor, they suppressed the growth of *F. nucleatum.* They increased the growth of *Clostridium butyricum* a bacterium that secretes butyrate and exhibits anti-tumor properties. Therefore, using phages to modify the gut microbiota may offer a novel approach to treating colorectal cancer [231]. Researchers used mouse models to explore the potential of genetically modified filamentous M13 bacteriophages as delivery vehicles for exogenous CRISPR-Cas9 DNA targeting *E. coli* in the gastrointestinal tract. The results showed chromosomal deletions in targeted bacteria both in vivo and in vitro after using the phages as a delivery method for CRISPR systems [230]. Another study developed engineered SNIPR001, a mixture of four phages modified with tail fibers and CRISPR-Cas machinery, to specifically target *E. coli*. Oral administration of a high-dose phage cocktail (2x10^^11^ PFU) was able to reduce *E. coli* levels in the guts of mice by 4 log CFU/g without harming the health of the animals or disrupting gut flora. A clinical trial is currently underway in the United States to assess the safety and effectiveness of this phage cocktail SNIPR001 against *E. coli* infections in humans with multiple oral doses [233].

## 11. Conclusion

The study of dark matter in gut microbiota is emerging thanks to advanced molecular and bioinformatic tools. We thoroughly examined the characteristics of the human and gut virome in both health and disease, as well as the complex relationships among intestinal bacteria, viruses or phages, and the human or animal host. The virome plays a role in several homeostatic processes and in regulating microbial populations through interactions with phages and bacteria. Many natural systems, such as marine environments, aquifer sediments, human infectious diseases, and animal intestinal ecosystems, show viral influences on microbes. Proper and stable gut virome configurations are crucial for maintaining a healthy gut microbiota. As a result, the field of viromics needs further development because viruses are both exciting and challenging. There is increasing evidence that gut bacteriophages significantly influence gut health, and our understanding of how specific viruses function in different ecosystems is evolving. Overcoming current challenges, like improving virome separation methods and expanding databases of uncultivated viruses, will be key to advancing our knowledge of the human gut virome.

### Limitation

Some unresolved issues need further investigation. The link between the gut virome and various illnesses remains mostly unknown regarding whether there is a correlation or causation, and virome research is limited by the lack of comprehensive reference databases and computational tools. The entire gut virome is significantly understudied, with over 50% of viral sequences identified through meta-genomic sequencing still without classification. This reduces the accuracy of current results and conclusions. Generally, less research has been conducted on the gut RNA virome compared to the DNA virome. Detailed studies on the molecular mechanisms of different intestinal viruses and phages that relate to host phenotypes are essential to develop next-generation microbe-based products aimed at improving human and animal health and treating specific diseases.

There is an increasing interest in studying the gut virome's function in aging, however research on this topic is still in its early stages and has a lot of problems with methods and ideas. A significant portion of the current information is obtained from animal models, predominantly mice, which, although informative, fail to completely represent the intricacies of the human gut microbiome or immune system [234]. Age-associated virome alterations identified in these models may not directly correlate with humans owing to interspecies variations in microbial composition, nutrition, longevity, and immunological function. Furthermore, investigations frequently depend on metagenomic sequencing, which may overlook low-abundance viral species and fail to differentiate between active and dormant phage populations. These technical constraints impede a thorough comprehension of the dynamic modulation of microbial communities by phages across time [91]

Furthermore, assertions about phage-induced microbial stability and immunological regulation in aging are often based on correlative evidence, lacking thorough analysis of causative relationships [235]. Few studies consider confounding factors like drug use, chronic inflammation, or common comorbidities in older populations, all of which can independently affect the virome. Similarly, early evidence for vertical transmission or transfer of virome components (e.g., through breast milk or early-life exposures) is still limited and often relies on small cohorts or single case studies[77]. It is difficult to determine whether observed changes in the virome are a cause or consequence of age-related physiological decline without large-scale, longitudinal human data. Additionally, ethical and logistical challenges make research on older adults, especially those who are vulnerable or in institutions, more difficult[236]. Therefore, although the idea of a phage-mediated mechanism for gut microbial stability and immune regulation in aging is intriguing, current evidence requires careful interpretation. There is an urgent need for more rigorous, human-focused studies to fill these knowledge gaps.

## 12. Future Perspective

Research on viruses has mainly concentrated on those that cause obvious diseases, leaving many unknowns about the numerous viruses that live within us 11many of which are yet to be identified. Some inflammatory diseases, especially those linked to industrialization or specific regions, may be affected by a changing virome, making it an important factor in how diseases develop. For complex conditions like Inflammatory Bowel Disease (IBD), there could be multiple pathways leading to the disease, with viruses being one of many contributing factors. If viruses do influence disease susceptibility, then several key objectives in virology become essential.

1. Cataloging the mammalian virome: To understand the diversity and prevalence of viruses in hosts, it is crucial to catalog the mammalian virome. Despite technological challenges, advances in deep sequencing and bioinformatics are making this increasingly achievable.
2. Establishing advanced *in vitro* infection models: Innovations in cell culture, like organoids and 3D cultures, will enable the study of viruses that are otherwise hard to examine. These models can be further improved by adding leukocytes and commensal bacteria to simulate multicellular and polymicrobial interactions.
3. Identifying Novel Viral Properties in Animal Models: Animal models, along with model viruses like LCMV and MNV, will continue to reveal previously unrecognized effects of viral immunomodulation and inter-kingdom interactions that only become clear in a living organism.
4. Including viruses in systems-level analyses: Quantifying multiple parameters in well-defined human and animal cohorts can reveal complex interactions between genetic and environmental factors. By studying the host, virus, and other microbiome members together, a more comprehensive understanding of mammalian biology can be achieved.

When exploring the virome, it’s crucial to remember that it includes many serious pathogens. Developing effective antivirals and vaccines will continue to be a priority, and evidence of beneficial viral effects should not impede these efforts. Instead, research should focus on understanding the factors and pathways that determine whether a virus harms or benefits the host. This deeper understanding could guide the development of immunomodulatory therapies for both viral and non-viral diseases.

Environmental Factors and the Gut Virome: The environment can greatly affect the composition and ecology of the gut virome, which, in turn, influences host health. Understanding these environmental factors could offer valuable insights into the gut virome's role in health and disease.

Gut Virome and Zoonotic Disease: The gut virome may also influence the spread of zoonotic diseases. Differences in the gut virome between species can impact how zoonotic diseases transmit across species, potentially posing risks to both animal and human health.

## References

[1] Cao Z, Sugimura N, Burgermeister E, Ebert MP, Zuo T, Lan P (2022). The gut virome: A new microbiome component in health and disease. EBioMedicine, 81. 10.1016/j.ebiom.2022.104113 PMC9240800 35753153

[2] Breitbart M, Hewson I, Felts B, Mahaffy JM, Nulton J, Salamon P, et al. (2003). Metagenomic analyses of an uncultured viral community from human feces. J Bacteriol, 185:6220-6223. 10.1128/JB.185.20.6220-6223.2003 PMC225035 14526037

[3] Minot S, Grunberg S, Wu GD, Lewis JD, Bushman FD (2012). Hypervariable loci in the human gut virome. Proc Natl Acad Sci, 109:3962-3966. 10.1073/pnas.1119061109 PMC3309749 22355105

[4] Wang H, Ling Y, Shan T, Yang S, Xu H, Deng X, et al. (2019). Gut virome of mammals and birds reveals high genetic diversity of the family Microviridae. Virus Evol, 5:vez013. 10.1093/ve/vez013 PMC6555873 31191981

[5] Santiago-Rodriguez TM, Hollister EB (2019). Human virome and disease: high-throughput sequencing for virus discovery, identification of phage-bacteria dysbiosis and development of therapeutic approaches with emphasis on the human gut. Viruses, 11:656. 10.3390/v11070656 PMC6669467 31323792

[6] Esposito AM, Esposito MM, Ptashnik A (2022). Phylogenetic diversity of animal oral and gastrointestinal viromes useful in surveillance of zoonoses. Microorganisms, 10:1815. 10.3390/microorganisms10091815 PMC9506515 36144417

[7] Xie XT, Kropinski AM, Tapscott B, Weese JS, Turner PV (2019). Prevalence of fecal viruses and bacteriophage in Canadian farmed mink (Neovison vison). MicrobiologyOpen, 8:e00622. 10.1002/mbo3.622 PMC6341152 29635866

[8] Barrientos-Somarribas M, Messina DN, Pou C, Lysholm F, Bjerkner A, Allander T, et al. (2018). Discovering viral genomes in human metagenomic data by predicting unknown protein families. Sci Rep, 8:28. 10.1038/s41598-017-18341-7 PMC5758519 29311716

[9] Adams MJ, Lefkowitz EJ, King AM, Harrach B, Harrison RL, Knowles NJ, et al. (2016). Ratification vote on taxonomic proposals to the International Committee on Taxonomy of Viruses (2016). Arch Virol, 161:2921-2949. 10.1007/s00705-016-2977-6 PMC7086986 27424026

[10] Brentlinger KL, Hafenstein S, Novak CR, Fane BA, Borgon R, McKenna R, et al. (2002). Microviridae, a family divided: isolation, characterization, and genome sequence of φMH2K, a bacteriophage of the obligate intracellular parasitic bacterium Bdellovibrio bacteriovorus. J Bacteriol, 184:1089-1094. 10.1128/jb.184.4.1089-1094.2002 PMC134817 11807069

[11] Minot S, Sinha R, Chen J, Li H, Keilbaugh SA, Wu GD, et al. (2011). The human gut virome: inter-individual variation and dynamic response to diet. Genome Res, 21:1616-1625. 10.1101/gr.122705.111 PMC3202279 21880779

[12] Reyes A, Haynes M, Hanson N, Angly FE, Heath AC, Rohwer F, et al. (2010). Viruses in the faecal microbiota of monozygotic twins and their mothers. Nature, 466:334-338. 10.1038/nature09199 PMC2919852 20631792

[13] Wang R, Bai B, Huang Y, Degen A, Mi J, Xue Y, et al. (2024). Yaks Are Dependent on Gut Microbiota for Survival in the Environment of the Qinghai Tibet Plateau. Microorganisms, 12. 10.3390/microorganisms12061122 PMC11205922 38930503

[14] Alsaaod M, Weber J, Jensen T, Brandt S, Gurtner C, Devaux D, et al. (2022). "Non-healing" claw horn lesions in dairy cows: Clinical, histopathological and molecular biological characterization of four cases. Front Vet Sci, 9:1041215. 10.3389/fvets.2022.1041215 PMC9627347 36337205

[15] Chaffringeon L, Lamy-Besnier Q, Debarbieux L, De Sordi L (2021). The intestinal virome: lessons from animal models. Curr Opin Virol, 51:141-148. 10.1016/j.coviro.2021.09.016 34700287

[16] Neil JA, Cadwell K (2018). The intestinal virome and immunity. J Immun, 201:1615-1624. 10.4049/jimmunol.1800631 PMC6179364 30181300

[17] Modi SR, Lee HH, Spina CS, Collins JJ (2013). Antibiotic treatment expands the resistance reservoir and ecological network of the phage metagenome. Nature, 499:219-222. 10.1038/nature12212 PMC3710538 23748443

[18] Gregory AC, Zablocki O, Zayed AA, Howell A, Bolduc B, Sullivan MB (2020). The gut virome database reveals age-dependent patterns of virome diversity in the human gut. Cell Host Microbe, 28:724-740. e728. 10.1016/j.chom.2020.08.003 PMC7443397 32841606

[19] Silveira CB, Rohwer FL (2016). Piggyback-the-Winner in host-associated microbial communities. npj Biofilms and Microbiomes, 2:1-5. 10.1038/npjbiofilms.2016.10 PMC5515262 28721247

[20] Pargin E, Roach MJ, Skye A, Papudeshi B, Inglis LK, Mallawaarachchi V, et al. (2023). The human gut virome: composition, colonization, interactions, and impacts on human health. Front Microbiol, 14:963173. 10.3389/fmicb.2023.963173 PMC10244655 37293229

[21] Barr JJ, Auro R, Furlan M, Whiteson KL, Erb ML, Pogliano J, et al. (2013). Bacteriophage adhering to mucus provide a non-host-derived immunity. Proc Natl Acad Sci, 110:10771-10776. 10.1073/pnas.1305923110 PMC3696810 23690590

[22] Shkoporov AN, Hill C (2019). Bacteriophages of the human gut: the “known unknown” of the microbiome. Cell Host Microbe, 25:195-209. 10.1016/j.chom.2019.01.017 30763534

[23] Liang G, Bushman FD (2021). The human virome: assembly, composition and host interactions. Nat Rev Microbiol, 19:514-527. 10.1038/s41579-021-00536-5 PMC8008777 33785903

[24] Zuo T, Sun Y, Wan Y, Yeoh YK, Zhang F, Cheung CP, et al. (2020). Human-gut-DNA virome variations across geography, ethnicity, and urbanization. Cell Host Microbe, 28:741-751. 10.1016/j.chom.2020.08.005 32910902

[25] Nakatsu G, Zhou H, Wu WKK, Wong SH, Coker OO, Dai Z, et al. (2018). Alterations in enteric virome are associated with colorectal cancer and survival outcomes. Gastroenterol, 155:529-541. 10.1053/j.gastro.2018.04.018 29689266

[26] Zhang T, Breitbart M, Lee WH, Run J-Q, Wei CL, Soh SWL, et al. (2006). RNA viral community in human feces: prevalence of plant pathogenic viruses. PLoS Biol, 4:e3. 10.1371/journal.pbio.0040003 PMC1310650 16336043

[27] Krishnamurthy SR, Janowski AB, Zhao G, Barouch D, Wang D (2016). Hyperexpansion of RNA bacteriophage diversity. PLoS Biol, 14:e1002409. 10.1371/journal.pbio.1002409 PMC4807089 27010970

[28] McCann A, Ryan FJ, Stockdale SR, Dalmasso M, Blake T, Ryan CA, et al. (2018). Viromes of one year old infants reveal the impact of birth mode on microbiome diversity. PeerJ, 6:e4694. 10.7717/peerj.4694 PMC5944432 29761040

[29] Baumann-Dudenhoeffer AM, D’Souza AW, Tarr PI, Warner BB, Dantas G (2018). Infant diet and maternal gestational weight gain predict early metabolic maturation of gut microbiomes. Nat Med, 24:1822-1829. 10.1038/s41591-018-0216-2 PMC6294307 30374198

[30] Duranti S, Lugli GA, Mancabelli L, Armanini F, Turroni F, James K, et al. (2017). Maternal inheritance of bifidobacterial communities and bifidophages in infants through vertical transmission. Microbiome, 5:1-13. 10.1186/s40168-017-0282-6 PMC5485682 28651630

[31] Liang G, Zhao C, Zhang H, Mattei L, Sherrill-Mix S, Bittinger K, et al. (2020). The stepwise assembly of the neonatal virome is modulated by breastfeeding. Nature, 581:470-474. 10.1038/s41586-020-2192-1 PMC7263352 32461640

[32] Desai C, Handley SA, Rodgers R, Rodriguez C, Ordiz MI, Manary MJ, et al. (2020). Growth velocity in children with Environmental Enteric Dysfunction is associated with specific bacterial and viral taxa of the gastrointestinal tract in Malawian children. PLoS Negl Trop Dis, 14:e0008387. 10.1371/journal.pntd.0008387 PMC7310680 32574158

[33] Sánchez B, Delgado S, Blanco-Míguez A, Lourenço A, Gueimonde M, Margolles A (2017). Probiotics, gut microbiota, and their influence on host health and disease. Mol Nutr Food Res, 61. 10.1002/mnfr.201600240 27500859

[34] Callanan J, Stockdale SR, Shkoporov A, Draper LA, Ross RP, Hill C (2020). Expansion of known ssRNA phage genomes: from tens to over a thousand. Sci Adv, 6:eaay5981. 10.1126/sciadv.aay5981 PMC7007245 32083183

[35] Nayfach S, Páez-Espino D, Call L, Low SJ, Sberro H, Ivanova NN, et al. (2021). Metagenomic compendium of 189,680 DNA viruses from the human gut microbiome. Nat Microbiol, 6:960-970. 10.1038/s41564-021-00928-6 PMC8241571 34168315

[36] Turner D, Kropinski AM, Adriaenssens EM (2021). A roadmap for genome-based phage taxonomy. Viruses, 13:506. 10.3390/v13030506 PMC8003253 33803862

[37] Koonin EV, Yutin N (2020). The crAss-like phage group: how metagenomics reshaped the human virome. Trends Microbiol, 28:349-359. 10.1016/j.tim.2020.01.010 32298613

[38] Minot S, Bryson A, Chehoud C, Wu GD, Lewis JD, Bushman FD (2013). Rapid evolution of the human gut virome. Proc Natl Acad Sci, 110:12450-12455. 10.1073/pnas.1300833110 PMC3725073 23836644

[39] James Cann A, Elizabeth Fandrich S, Heaphy S (2005). Analysis of the virus population present in equine faeces indicates the presence of hundreds of uncharacterized virus genomes. PLoS Negl Trop Dis, 30:151-156. 10.1007/s11262-004-5624-3 15744573

[40] Ng TFF, Mesquita JR, Nascimento MSJ, Kondov NO, Wong W, Reuter G, et al. (2014). Feline fecal virome reveals novel and prevalent enteric viruses. Vet Microbiol, 171:102-111. 10.1016/j.vetmic.2014.04.005 PMC4080910 24793097

[41] Zhang B, Chassaing B, Shi Z, Uchiyama R, Zhang Z, Denning TL, et al. (2014). Prevention and cure of rotavirus infection via TLR5/NLRC4-mediated production of IL-22 and IL-18. Science, 346:861-865. 10.1126/science.1256999 PMC4788408 25395539

[42] Shi Y, Tao J, Li B, Shen X, Cheng J, Liu H (2021). The Gut Viral Metagenome Analysis of Domestic Dogs Captures Snapshot of Viral Diversity and Potential Risk of Coronavirus. Front Vet Sci, 8:695088. 10.3389/fvets.2021.695088 PMC8292670 34307533

[43] Li Y, Altan E, Reyes G, Halstead B, Deng X, Delwart E (2021). Virome of bat guano from nine Northern California roosts. Virol J, 95:10.1128/jvi.01713-01720. PMC7925108 33115864

[44] Amimo JO, El Zowalaty ME, Githae D, Wamalwa M, Djikeng A, Nasrallah GK (2016). Metagenomic analysis demonstrates the diversity of the fecal virome in asymptomatic pigs in East Africa. Arch Virol, 161:887-897. 10.1007/s00705-016-2819-6 26965436

[45] Varsani A, Krupovic M (2018). Smacoviridae: a new family of animal-associated single-stranded DNA viruses., Arch Virol, 163:2005-2015. 10.1007/s00705-018-3820-z 29572596

[46] Chen Q, Wang L, Zheng Y, Zhang J, Guo B, Yoon K-J, et al. (2018). Metagenomic analysis of the RNA fraction of the fecal virome indicates high diversity in pigs infected by porcine endemic diarrhea virus in the United States. Virol J, 15:1-9. 10.1186/s12985-018-1001-z PMC5970503 29801460

[47] Shan T, Yang S, Wang H, Wang H, Zhang J, Gong G, et al. (2022). Virome in the cloaca of wild and breeding birds revealed a diversity of significant viruses. Microbiome, 10:60. 10.1186/s40168-022-01246-7 PMC9001828 35413940

[48] Cai J, Sun L, Gonzalez FJ (2022). Gut microbiota-derived bile acids in intestinal immunity, inflammation, and tumorigenesis. Cell Host Microbe, 30:289-300. 10.1016/j.chom.2022.02.004 PMC8923532 35271802

[49] Duan Y, Llorente C, Lang S, Brandl K, Chu H, Jiang L, et al. (2019). Bacteriophage targeting of gut bacterium attenuates alcoholic liver disease. Nature, 575:505-511. 10.1038/s41586-019-1742-x PMC6872939 31723265

[50] Cepko LC, Garling EE, Dinsdale MJ, Scott WP, Bandy L, Nice T, et al. (2020). Myoviridae phage PDX kills enteroaggregative Escherichia coli without human microbiome dysbiosis. J Med Microbiol, 69:309-323. 10.1099/jmm.0.001162 PMC7431101 32011231

[51] Harvey E, Holmes EC (2022). Diversity and evolution of the animal virome. Nat Rev Microbiol, 20:321-334. 10.1038/s41579-021-00665-x 34983966

[52] Reyes A, Blanton LV, Cao S, Zhao G, Manary M, Trehan I, et al. (2015). Gut DNA viromes of Malawian twins discordant for severe acute malnutrition. Proc Natl Acad Sci, 112:11941-11946. 10.1073/pnas.1514285112 PMC4586842 26351661

[53] Kim MS, Bae JW (2016). Spatial disturbances in altered mucosal and luminal gut viromes of diet-induced obese mice. Environ Microbiol, 18:1498-1510. 10.1111/1462-2920.13182 26690305

[54] Zhu H, Wen Q, Bhangu SK, Ashokkumar M, Cavalieri F (2022). Sonosynthesis of nanobiotics with antimicrobial and antioxidant properties. Ultrasonics Sonochemistry, 86:106029. 10.1016/j.ultsonch.2022.106029 PMC9112028 35561593

[55] Sauvage V, Cheval J, Foulongne V, Gouilh MA, Pariente K, Manuguerra JC, et al. (2011). Identification of the first human gyrovirus, a virus related to chicken anemia virus. Virol J, 85:7948-7950. 10.1128/JVI.00639-11 PMC3147908 21632766

[56] Phan TG, Li L, O’Ryan MG, Cortes H, Mamani N, Bonkoungou IJ, et al. (2012). A third gyrovirus species in human faeces. J Gen Virol, 93:1356-1361. 10.1099/vir.0.041731-0 PMC3755516 22422066

[57] Chu DK, Poon LL, Chiu SS, Chan KH, Ng EM, Bauer I, et al. (2012). Characterization of a novel gyrovirus in human stool and chicken meat. J Clin Virol, 55:209-213. 10.1016/j.jcv.2012.07.001 PMC3449218 22824231

[58] Columpsi P, Sacchi P, Zuccaro V, Cima S, Sarda C, Mariani M, et al. (2016). Beyond the gut bacterial microbiota: The gut virome. J Med Virol, 88:1467-1472. 10.1002/jmv.24508 PMC7166815 26919534

[59] Delwart E, Li L (2012). Rapidly expanding genetic diversity and host range of the Circoviridae viral family and other Rep encoding small circular ssDNA genomes. Virus Res, 164:114-121. 10.1016/j.virusres.2011.11.021 PMC3289258 22155583

[60] Tamura K, Dudley J, Nei M, Kumar S (2007). MEGA4: molecular evolutionary genetics analysis (MEGA) software version 4.0. Mol Biol Evol, 24:1596-1599. 10.1093/molbev/msm092 17488738

[61] Weinbauer MG (2004). Ecology of prokaryotic viruses. FEMS Microbiol Rev, 28:127-181. 10.1016/j.femsre.2003.08.001 15109783

[62] Wilhelm SW, Suttle CA (1999). Viruses and nutrient cycles in the sea: viruses play critical roles in the structure and function of aquatic food webs. Biosci, 49:781-788.

[63] Rohwer F, Prangishvili D, Lindell D. 2009. Roles of viruses in the environment. Wiley Online Library. 2771-2774. 10.1111/j.1462-2920.2009.02101.x 19878268

[64] Ley RE, Peterson DA, Gordon JI (2006). Ecological and evolutionary forces shaping microbial diversity in the human intestine. Cell, 124:837-848. 10.1016/j.cell.2006.02.017 16497592

[65] Turnbaugh PJ, Ley RE, Hamady M, Fraser-Liggett CM, Knight R, Gordon JI (2007). The human microbiome project. Nature, 449:804-810. 10.1038/nature06244 PMC3709439 17943116

[66] Frank DN, St. Amand AL, Feldman RA, Boedeker EC, Harpaz N, Pace NR (2007). Molecular-phylogenetic characterization of microbial community imbalances in human inflammatory bowel diseases. Proc Natl Acad Sci, 104:13780-13785. 10.1073/pnas.0706625104 PMC1959459 17699621

[67] Wen L, Ley RE, Volchkov PY, Stranges PB, Avanesyan L, Stonebraker AC, et al. (2008). Innate immunity and intestinal microbiota in the development of Type 1 diabetes. Nature, 455:1109-1113. 10.1038/nature07336 PMC2574766 18806780

[68] Qin J, Li R, Raes J, Arumugam M, Burgdorf KS, Manichanh C, et al. (2010). A human gut microbial gene catalogue established by metagenomic sequencing. Nature, 464:59-65. 10.1038/nature08821 PMC3779803 20203603

[69] Roberts AP, Chandler M, Courvalin P, Guédon G, Mullany P, Pembroke T, et al. (2008). Revised nomenclature for transposable genetic elements. Plasmid, 60:167-173. 10.1016/j.plasmid.2008.08.001 PMC3836210 18778731

[70] Tamames J, Moya A (2008). Estimating the extent of horizontal gene transfer in metagenomic sequences. BMC genomics, 9:1-15. 10.1186/1471-2164-9-136 PMC2324111 18366724

[71] Delwart EL (2007). Viral metagenomics. Rev Med Virol, 17:115-131. 10.1002/rmv.532 PMC7169062 17295196

[72] Moreno-Gallego JL, Chou S-P, Di Rienzi SC, Goodrich JK, Spector TD, Bell JT, et al. (2019). Virome diversity correlates with intestinal microbiome diversity in adult monozygotic twins. Cell Host Microbe, 25:261-272. e265. 10.1016/j.chom.2019.01.019 PMC6411085 30763537

[73] Ogilvie LA, Bowler LD, Caplin J, Dedi C, Diston D, Cheek E, et al. (2013). Genome signature-based dissection of human gut metagenomes to extract subliminal viral sequences., Nat commun, 4:2420. 10.1038/ncomms3420 PMC3778543 24036533

[74] Camarillo-Guerrero LF, Almeida A, Rangel-Pineros G, Finn RD, Lawley TD (2021). Massive expansion of human gut bacteriophage diversity. Cell, 184:1098-1109. e1099. 10.1016/j.cell.2021.01.029 PMC7895897 33606979

[75] Li J, Jia H, Cai X, Zhong H, Feng Q, Sunagawa S, et al. (2014). An integrated catalog of reference genes in the human gut microbiome. Nat Biotechnol, 32:834-841. 10.1038/nbt.2942 24997786

[76] Beller L, Matthijnssens J (2019). What is (not) known about the dynamics of the human gut virome in health and disease. Curr Opin Virol, 37:52-57. 10.1016/j.coviro.2019.05.013 31255903

[77] Lim ES, Zhou Y, Zhao G, Bauer IK, Droit L, Ndao IM, et al. (2015). Early life dynamics of the human gut virome and bacterial microbiome in infants Nat. Med, 21:1228-1234. 10.1038/nm.3950 PMC4710368 26366711

[78] Cryan JF, O'Riordan KJ, Cowan CS, Sandhu KV, Bastiaanssen TF, Boehme M, et al. (2019). The microbiota-gut-brain axis. Physiol Rev, 99:1877-2013 10.1152/physrev.00018.2018 31460832

[79] Mukhopadhya I, Segal JP, Carding SR, Hart AL, Hold GL (2019). The gut virome: the ‘missing link’between gut bacteria and host immunity? Therap Adv Gastroenterol, 12:1756284819836620. 10.1177/1756284819836620 PMC6435874 30936943

[80] Fulci V, Stronati L, Cucchiara S, Laudadio I, Carissimi C (2021). Emerging roles of gut virome in pediatric diseases. Int J Mol Sci, 22:4127. 10.3390/ijms22084127 PMC8073368 33923593

[81] Hopkins M, Kailasan S, Cohen A, Roux S, Tucker KP, Shevenell A, et al. (2014). Diversity of environmental single-stranded DNA phages revealed by PCR amplification of the partial major capsid protein. The ISME journal, 8:2093-2103. 10.1038/ismej.2014.43 PMC4184009 24694711

[82] Gogokhia L, Buhrke K, Bell R, Hoffman B, Brown DG, Hanke-Gogokhia C, et al. (2019). Expansion of bacteriophages is linked to aggravated intestinal inflammation and colitis. Cell Host Microbe, 25:285-299. e288. 10.1016/j.chom.2019.01.008 PMC6885004 30763538

[83] Ezra-Nevo G, Henriques SF, Ribeiro C (2020). The diet-microbiome tango: how nutrients lead the gut brain axis. Curr Opin Neurobiol, 62:122-132. 10.1016/j.conb.2020.02.005 32199342

[84] Broecker F, Klumpp J, Schuppler M, Russo G, Biedermann L, Hombach M, et al. (2016). Long-term changes of bacterial and viral compositions in the intestine of a recovered Clostridium difficile patient after fecal microbiota transplantation. Mol Case Stud, 2:a000448. 10.1101/mcs.a000448 PMC4849847 27148577

[85] Norman JM, Handley SA, Baldridge MT, Droit L, Liu CY, Keller BC, et al. (2015). Disease-specific alterations in the enteric virome in inflammatory bowel disease. Cell, 160:447-460. 10.1016/j.cell.2015.01.002 PMC4312520 25619688

[86] Zuo T, Lu X-J, Zhang Y, Cheung CP, Lam S, Zhang F, et al. (2019). Gut mucosal virome alterations in ulcerative colitis. Gut, 68:1169-1179. 10.1136/gutjnl-2018-318131 PMC6582748 30842211

[87] Broecker F, Klumpp J, Moelling K (2016). Long-term microbiota and virome in a Zürich patient after fecal transplantation against Clostridium difficile infection. Ann N Y Acad Sci, 1372:29-41. 10.1111/nyas.13100 27286042

[88] Park A, Zhao G (2018). Mining the virome for insights into type 1 diabetes. Dna Cell Biol, 37:422-425. 10.1089/dna.2018.4185 PMC5944395 29638163

[89] Cheng L, Qi C, Zhuang H, Fu T, Zhang X (2020). gutMDisorder: a comprehensive database for dysbiosis of the gut microbiota in disorders and interventions. Nucleic Acids Res, 48:D554-D560. 10.1093/nar/gkz843 PMC6943049 31584099

[90] Zuo T, Kamm MA, Colombel J-F, Ng SC (2018). Urbanization and the gut microbiota in health and inflammatory bowel disease. Nat Rev Gastroenterol Hepatol, 15:440-452. 10.1038/s41575-018-0003-z 29670252

[91] Liang G, Bushman FD (2021). The human virome: assembly, composition and host interactions. Nat Rev Microbiol, 19:514-527. 10.1038/s41579-021-00536-5 PMC8008777 33785903

[92] Sutton TD, Hill C (2019). Gut bacteriophage: current understanding and challenges. Front Endocrinol, 10:784. 10.3389/fendo.2019.00784 PMC6895007 31849833

[93] Duerkop BA (2018). Bacteriophages shift the focus of the mammalian microbiota. PLoS Pathog, 14:e1007310. 10.1371/journal.ppat.1007310 PMC6201943 30359456

[94] Ramanan D, Cadwell K (2016). Intrinsic defense mechanisms of the intestinal epithelium. Cell Host Microbe, 19:434-441. 10.1016/j.chom.2016.03.003 PMC4861060 27049583

[95] Federici S, Nobs SP, Elinav E (2021). Phages and their potential to modulate the microbiome and immunity. Cell Mol Immunol, 18:889-904. 10.1038/s41423-020-00532-4 PMC8115240 32901128

[96] Korkina L, Ozben T, Saso L. (2016). Modulation of oxidative stress: pharmaceutical and pharmacological aspects. Oxid Med Cell Longev, 6023417. 10.1155/2016/6023417 PMC4783544 27006747

[97] Yue WanFu YW, Du Min DM, Zhu MeiJun ZM (2012). High temperature in combination with UV irradiation enhances horizontal transfer of stx 2 gene from E. coli O157: H7 to non-pathogenic E. coli. PLoS One, 7:e31308 10.1371/journal.pone.0031308 PMC3276539 22347461

[98] Boling L, Cuevas DA, Grasis JA, Kang HS, Knowles B, Levi K, et al. (2020). Dietary prophage inducers and antimicrobials: toward landscaping the human gut microbiome. Gut Microbes, 11:721-734. 10.1080/19490976.2019.1701353 PMC7524278 31931655

[99] Liu X, Lin S, Liu T, Zhou Y, Wang W, Yao J, et al. (2021). Xenogeneic silencing relies on temperature-dependent phosphorylation of the host H-NS protein in Shewanella. Nucleic Acids Res, 49:3427-3440. 10.1093/nar/gkab137 PMC8034616 33693785

[100] Łoś M, Węgrzyn G (2012). Pseudolysogeny. ADV VIRUS RES 82:339-349. 10.1016/B978-0-12-394621-8.00019-4 22420857

[101] Buchmann JP, Holmes EC (2015). Cell walls and the convergent evolution of the viral envelope. Microbiol Mol Biol Rev, 79:403-418. 10.1128/MMBR.00017-15 PMC4651029 26378223

[102] De Paepe M, Leclerc M, Tinsley CR, Petit M-A (2014). Bacteriophages: an underestimated role in human and animal health? Front Cell Infect Microbiol, 4:39. 10.3389/fcimb.2014.00039 PMC3975094 24734220

[103] Reyes A, Wu M, McNulty NP, Rohwer FL, Gordon JI (2013). Gnotobiotic mouse model of phage-bacterial host dynamics in the human gut. Proc Natl Acad Sci, 110:20236-20241. 10.1073/pnas.1319470110 PMC3864308 24259713

[104] Duerkop BA, Clements CV, Rollins D, Rodrigues JL, Hooper LV (2012). A composite bacteriophage alters colonization by an intestinal commensal bacterium. Proc Natl Acad Sci, 109:17621-17626. 10.1073/pnas.1206136109 PMC3491505 23045666

[105] Mills S, Shanahan F, Stanton C, Hill C, Coffey A, Ross RP (2013). Movers and shakers: influence of bacteriophages in shaping the mammalian gut microbiota. Gut Microbes, 4:4-16. 10.4161/gmic.22371 PMC3555884 23022738

[106] Hernandez-Doria JD, Sperandio V (2018). Bacteriophage transcription factor Cro regulates virulence gene expression in enterohemorrhagic Escherichia coli. Cell Host Microbe, 23:607-617. 10.1016/j.chom.2018.04.007 PMC5982111 29746832

[107] Veses-Garcia M, Liu X, Rigden DJ, Kenny JG, McCarthy AJ, Allison HE (2015). Transcriptomic analysis of Shiga-toxigenic bacteriophage carriage reveals a profound regulatory effect on acid resistance in Escherichia coli. Appl Environ Microbiol, 81:8118-8125. 10.1128/AEM.02034-15 PMC4651098 26386055

[108] Barr JJ, Auro R, Sam-Soon N, Kassegne S, Peters G, Bonilla N, et al. (2015). Subdiffusive motion of bacteriophage in mucosal surfaces increases the frequency of bacterial encounters. Proc Natl Acad Sci, 112:13675-13680. 10.1073/pnas.1508355112 PMC4640763 26483471

[109] Varga M, Kuntová L, Pantůček R, Mašlaňová I, Růžičková V, Doškař J (2012). Efficient transfer of antibiotic resistance plasmids by transduction within methicillin-resistant Staphylococcus aureus USA300 clone. FEMS Microbiol Lett, 332:146-152. 10.1111/j.1574-6968.2012.02589.x 22553940

[110] Goh S, Hussain H, Chang BJ, Emmett W, Riley TV, Mullany P (2013). Phage ϕC2 mediates transduction of Tn 6215, encoding erythromycin resistance, between Clostridium difficile strains. MBio, 4:10.1128/mbio.00840-00813. PMC3870246 24255122

[111] Mazaheri Nezhad Fard R, Barton M, Heuzenroeder M (2011). Bacteriophage-mediated transduction of antibiotic resistance in enterococci. Lett. Appl. Microbiol, 52:559-564. 10.1111/j.1472-765X.2011.03043.x 21395627

[112] Enault F, Briet A, Bouteille L, Roux S, Sullivan MB, Petit M-A (2017). Phages rarely encode antibiotic resistance genes: a cautionary tale for virome analyses. The ISME journal, 11:237-247. 10.1038/ismej.2016.90 PMC5315482 27326545

[113] Billaud M, Lamy-Besnier Q, Lossouarn J, Moncaut E, Dion MB, Moineau S, et al. (2021). Analysis of viromes and microbiomes from pig fecal samples reveals that phages and prophages rarely carry antibiotic resistance genes. ISME Commun, 1:55. 10.1038/s43705-021-00054-8 PMC9723715 37938642

[114] Pennetzdorfer N, Popescu MC, Haddock NL, Dupuy F, Kaber G, Hargil A, et al. (2023). Bacterial outer membrane vesicles bound to bacteriophages modulate neutrophil responses to bacterial infection. Front Cell Infect Microbiol, 13:1250339. 10.3389/fcimb.2023.1250339 PMC10641230 37965262

[115] Brown EM, Arellano-Santoyo H, Temple ER, Costliow ZA, Pichaud M, Hall AB, et al. (2021). Gut microbiome ADP-ribosyltransferases are widespread phage-encoded fitness factors. Cell Host Microbe, 29:1351-1365. e1311. 10.1016/j.chom.2021.07.011 PMC8429246 34403684

[116] Aktories K, Schwan C, Jank T (2017). Clostridium difficile toxin biology. Annu Rev Microbiol, 71:281-307. 10.1146/annurev-micro-090816-093458 28657883

[117] Barr JJ, Auro R, Sam-Soon N, Kassegne S, Peters G, Bonilla N, et al. (2015). Subdiffusive motion of bacteriophage in mucosal surfaces increases the frequency of bacterial encounters. Proc Natl Acad Sci, 112:13675-13680. 10.1073/pnas.1508355112 PMC4640763 26483471

[118] Bichet MC, Chin WH, Richards W, Lin Y-W, Avellaneda-Franco L, Hernandez CA, et al. (2021). Bacteriophage uptake by mammalian cell layers represents a potential sink that may impact phage therapy. Iscience, 24. 10.1016/j.isci.2021.102287 PMC8024918 33855278

[119] Dąbrowska K (2019). Phage therapy: What factors shape phage pharmacokinetics and bioavailability? Systematic and critical review. Med Res Rev, 39:2000-2025. 10.1002/med.21572 PMC6767042 30887551

[120] Dery KJ, Górski A, Miedzybrodzki R, Farmer DG, Kupiec-Weglinski JW (2021). Therapeutic perspectives and mechanistic insights of phage therapy in allotransplantation. Transplant, 105:1449-1458. 10.1097/TP.0000000000003565 33273319

[121] Metzger RN, Krug AB, Eisenächer K (2018). Enteric virome sensing—its role in intestinal homeostasis and immunity. Viruses, 10:146. 10.3390/v10040146 PMC5923440 29570694

[122] Broquet AH, Hirata Y, McAllister CS, Kagnoff MF (2011). RIG-I/MDA5/MAVS are required to signal a protective IFN response in rotavirus-infected intestinal epithelium. J Immunol, 186:1618-1626. 10.4049/jimmunol.1002862 21187438

[123] Liu L, Gong T, Tao W, Lin B, Li C, Zheng X, et al. (2019). Commensal viruses maintain intestinal intraepithelial lymphocytes via noncanonical RIG-I signaling. Nat Immunol, 20:1681-1691. 10.1038/s41590-019-0513-z 31636462

[124] Abt MC, Buffie CG, Sušac B, Becattini S, Carter RA, Leiner I, et al. (2016). TLR-7 activation enhances IL-22-mediated colonization resistance against vancomycin-resistant enterococcus. Sci Transl Med, 8:327ra325-327ra325. 10.1126/scitranslmed.aad6663 PMC4991618 26912904

[125] Neil JA, Matsuzawa-Ishimoto Y, Kernbauer-Hölzl E, Schuster SL, Sota S, Venzon M, et al. (2019). IFN-I and IL-22 mediate protective effects of intestinal viral infection. Nat Microbiol, 4:1737-1749. 10.1038/s41564-019-0470-1 PMC6871771 31182797

[126] Ingle H, Lee S, Ai T, Orvedahl A, Rodgers R, Zhao G, et al. (2019). Viral complementation of immunodeficiency confers protection against enteric pathogens via interferon-λ. Nat Microbiol, 4:1120-1128. 10.1038/s41564-019-0416-7 PMC6588490 30936486

[127] Ott SJ, Waetzig GH, Rehman A, Moltzau-Anderson J, Bharti R, Grasis JA, et al. (2017). Efficacy of sterile fecal filtrate transfer for treating patients with Clostridium difficile infection. Gastroenterol, 152:799-811. e797. 10.1053/j.gastro.2016.11.010 27866880

[128] Zuo T, Wong SH, Lam K, Lui R, Cheung K, Tang W, et al. (2018). Bacteriophage transfer during faecal microbiota transplantation in Clostridium difficile infection is associated with treatment outcome. Gut, 67:634-643. 10.1136/gutjnl-2017-313952 PMC5868238 28539351

[129] Wang P, Zhu S, Yang L, Cui S, Pan W, Jackson R, et al. (2015). Nlrp6 regulates intestinal antiviral innate immunity. Science, 350:826-830. 10.1126/science.aab3145 PMC4927078 26494172

[130] Ingle H, Peterson ST, Baldridge MT (2018). Distinct effects of type I and III interferons on enteric viruses. Viruses, 10:46. 10.3390/v10010046 PMC5795459 29361691

[131] Hernández PP, Mahlakõiv T, Yang I, Schwierzeck V, Nguyen N, Guendel F, et al. (2015). Interferon-λ and interleukin 22 act synergistically for the induction of interferon-stimulated genes and control of rotavirus infection. Carbohydr Polym, 16:698-707. 10.1038/ni.3180 PMC4589158 26006013

[132] Yokoyama CC, Loh J, Zhao G, Stappenbeck TS, Wang D, Huang HV, et al. (2012). Adaptive immunity restricts replication of novel murine astroviruses. Virol J, 86:12262-12270. 10.1128/JVI.02018-12 PMC3486451 22951832

[133] Virgin HW (2014). The virome in mammalian physiology and disease. Cell, 157:142-150. 10.1016/j.cell.2014.02.032 PMC3977141 24679532

[134] Barr JJ, Auro R, Furlan M, Whiteson KL, Erb ML, Pogliano J, et al. (2013). Bacteriophage adhering to mucus provide a non-host-derived immunity. Proc Natl Acad Sci, 110:10771-10776. 10.1073/pnas.1305923110 PMC3696810 23690590

[135] Kaur S, Harjai K, Chhibber S (2014). Bacteriophage-aided intracellular killing of engulfed methicillin-resistant Staphylococcus aureus (MRSA) by murine macrophages. Appl Microbiol Biotechnol, 98:4653-4661. 10.1007/s00253-014-5643-5 24633444

[136] Fröhlich EE, Farzi A, Mayerhofer R, Reichmann F, Jačan A, Wagner B, et al. (2016). Cognitive impairment by antibiotic-induced gut dysbiosis: analysis of gut microbiota-brain communication. Brain Behav Immun, 56:140-155. 10.1016/j.bbi.2016.02.020 PMC5014122 26923630

[137] Handley SA, Thackray LB, Zhao G, Presti R, Miller AD, Droit L, et al. (2012). Pathogenic simian immunodeficiency virus infection is associated with expansion of the enteric virome. Cell, 151:253-266. 10.1016/j.cell.2012.09.024 PMC3490196 23063120

[138] Cadwell K (2015). The virome in host health and disease. Immunity, 42:805-813. 10.1016/j.immuni.2015.05.003 PMC4578625 25992857

[139] Rouse BT, Sehrawat S (2010). Immunity and immunopathology to viruses: what decides the outcome? Nat Rev Immunol, 10:514-526. 10.1038/nri2802 PMC3899649 20577268

[140] Yu Y, Wang W, Zhang F (2023). The Next Generation Fecal Microbiota Transplantation: To Transplant Bacteria or Virome. Adv Sci, 10:2301097. 10.1002/advs.202301097 PMC10724401 37914662

[141] Emencheta SC, Olovo CV, Eze OC, Kalu CF, Berebon DP, Onuigbo EB, et al. (2023). The Role of Bacteriophages in the Gut Microbiota: Implications for Human Health. Pharm., 15:2416. 10.3390/pharmaceutics15102416 PMC10609668 37896176

[142] Mayneris-Perxachs J, Castells-Nobau A, Arnoriaga-Rodríguez M, Garre-Olmo J, Puig J, Ramos R, et al. (2022). Caudovirales bacteriophages are associated with improved executive function and memory in flies, mice, and humans. Cell host & microbe, 30:340-356. e348. 10.1016/j.chom.2022.01.013 35176247

[143] Herskovits AA, Auerbuch V, Portnoy DA (2007). Bacterial ligands generated in a phagosome are targets of the cytosolic innate immune system. PLoS Pathog, 3:e51. 10.1371/journal.ppat.0030051 PMC1839167 17397264

[144] Barton ES, White DW, Cathelyn JS, Brett-McClellan KA, Engle M, Diamond MS, et al. (2007). Herpesvirus latency confers symbiotic protection from bacterial infection. Nature, 447:326-329. 10.1038/nature05762 17507983

[145] White DW, Keppel CR, Schneider SE, Reese TA, Coder J, Payton JE, et al. (2010). Latent herpesvirus infection arms NK cells. Blood, 115:4377-4383. 10.1182/blood-2009-09-245464 PMC2881492 20139098

[146] Thépaut M, Grandjean T, Hober D, Lobert P-E, Bortolotti P, Faure K, et al. (2015). Protective role of murine norovirus against Pseudomonas aeruginosa acute pneumonia. Vet Res, 46:1-7. 10.1186/s13567-015-0239-3 PMC4558952 26338794

[147] Kernbauer E, Ding Y, Cadwell K (2014). An enteric virus can replace the beneficial function of commensal bacteria. Nature, 516:94-98. 10.1038/nature13960 PMC4257755 25409145

[148] Machiels B, Dourcy M, Xiao X, Javaux J, Mesnil C, Sabatel C, et al. (2017). A gammaherpesvirus provides protection against allergic asthma by inducing the replacement of resident alveolar macrophages with regulatory monocytes. Nat Immunol, 18:1310-1320. 10.1038/ni.3857 29035391

[149] Duerkop BA, Kleiner M, Paez-Espino D, Zhu W, Bushnell B, Hassell B, et al. (2018). Murine colitis reveals a disease-associated bacteriophage community. Nat Microbiol, 3:1023-1031. 10.1038/s41564-018-0210-y PMC6112176 30038310

[150] Bao S, Wang H, Li W, Ji L, Wang X, Shen Q, et al. (2022). Dynamic alterations of the mice gut virome after Coxsackievirus B3 infection. J Med Virol, 94:4959-4969. 10.1002/jmv.27946 35718835

[151] Jernigan JA, Hatfield KM, Wolford H, Nelson RE, Olubajo B, Reddy SC, et al. (2020). Multidrug-resistant bacterial infections in US hospitalized patients, 2012-2017. N Engl J Med, 382:1309-1319. 10.1056/NEJMoa1914433 PMC10961699 32242356

[152] Flynn S, Eisenstein S (2019). Inflammatory bowel disease presentation and diagnosis. Surg Clin, 99:1051-1062. 10.1016/j.suc.2019.08.001 31676047

[153] Seyedian SS, Nokhostin F, Malamir MD (2019). A review of the diagnosis, prevention, and treatment methods of inflammatory bowel disease. J Life Med, 12:113. 10.25122/jml-2018-0075 PMC6685307 31406511

[154] Khan I, Ullah N, Zha L, Bai Y, Khan A, Zhao T, et al. (2019). Alteration of gut microbiota in inflammatory bowel disease (IBD): cause or consequence? IBD treatment targeting the gut microbiome. Pathog, 8:126. 10.3390/pathogens8030126 PMC6789542 31412603

[155] Kong C, Liu G, Kalady MF, Jin T, Ma Y (2023). Dysbiosis of the stool DNA and RNA virome in Crohn's disease. J Med Virol, 95:e28573. 10.1002/jmv.28573 36772850

[156] Clooney AG, Sutton TD, Shkoporov AN, Holohan RK, Daly KM, O’Regan O, et al. (2019). Whole-virome analysis sheds light on viral dark matter in inflammatory bowel disease. Cell Host Microbe, 26:764-778. e765. 10.1016/j.chom.2019.10.009 31757768

[157] Imai T, Inoue R, Nishida A, Yokota Y, Morishima S, Kawahara M, et al. (2022). Features of the gut prokaryotic virome of Japanese patients with Crohn’s disease. J Gastroenterol, 57:559-570. 10.1007/s00535-022-01882-8 35689701

[158] Lepage P, Colombet J, Marteau P, Sime-Ngando T, Doré J, Leclerc M (2008). Dysbiosis in inflammatory bowel disease: a role for bacteriophages? Gut, 57:424-425. 10.1136/gut.2007.134668 18268057

[159] Wang W, Jovel J, Halloran B, Wine E, Patterson J, Ford G, et al. (2015). Metagenomic analysis of microbiome in colon tissue from subjects with inflammatory bowel diseases reveals interplay of viruses and bacteria. Inflamm Bowel Dis, 21:1419-1427. 10.1097/MIB.0000000000000344 PMC4450971 25939040

[160] Lopes S, Andrade P, Conde S, Liberal R, Dias CC, Fernandes S, et al. (2017). Looking into enteric virome in patients with IBD: defining guilty or innocence? Inflamm Bowel Dis, 23:1278-1284. 10.1097/MIB.0000000000001167 28617757

[161] Ungaro F, Massimino L, Furfaro F, Rimoldi V, Peyrin-Biroulet L, D’alessio S, et al. (2019). Metagenomic analysis of intestinal mucosa revealed a specific eukaryotic gut virome signature in early-diagnosed inflammatory bowel disease. Gut Microbes, 10:149-158. 10.1080/19490976.2018.1511664 PMC6546319 30252582

[162] Younis M, Rastogi R, Chugh A, Rastogi S, Aly H (2020). Congenital diarrheal diseases. Clin Perinatol, 47:301-321. 10.1016/j.clp.2020.02.007 32439113

[163] Julio-Pieper M, López-Aguilera A, Eyzaguirre-Velásquez J, Olavarría-Ramírez L, Ibacache-Quiroga C, Bravo JA, et al. (2021). Gut susceptibility to viral invasion: contributing roles of diet, microbiota and enteric nervous system to mucosal barrier preservation. Int J Mol Sci, 22:4734. 10.3390/ijms22094734 PMC8125429 33946994

[164] Diard M, Bakkeren E, Cornuault JK, Moor K, Hausmann A, Sellin ME, et al. (2017). Inflammation boosts bacteriophage transfer between Salmonella spp. Science, 355:1211-1215. 10.1126/science.aaf8451 28302859

[165] Monaco CL, Gootenberg DB, Zhao G, Handley SA, Ghebremichael MS, Lim ES, et al. (2016). Altered virome and bacterial microbiome in human immunodeficiency virus-associated acquired immunodeficiency syndrome. Cell Host Microbe, 19:311-322. 10.1016/j.chom.2016.02.011 PMC4821831 26962942

[166] Brenchley JM, Douek D (2008). HIV infection and the gastrointestinal immune system. Mucosal Immunol, 1:23-30. 10.1038/mi.2007.1 PMC2777614 19079157

[167] Li L, Deng X, Da Costa AC, Bruhn R, Deeks SG, Delwart E (2015). Virome analysis of antiretroviral-treated HIV patients shows no correlation between T-cell activation and anelloviruses levels. J Clin Virol, 72:106-113. 10.1016/j.jcv.2015.09.004 PMC4697764 26479202

[168] Li L, Deng X, Linsuwanon P, Bangsberg D, Bwana MB, Hunt P, et al. (2013). AIDS alters the commensal plasma virome. Virol J, 87:10912-10915. 10.1128/JVI.01839-13 PMC3807392 23903845

[169] Siqueira JD, Curty G, Xutao D, Hofer CB, Machado ES, Seuánez HN, et al. (2019). Composite analysis of the virome and bacteriome of HIV/HPV co-infected women reveals proxies for immunodeficiency. Viruses, 11:422. 10.3390/v11050422 PMC6563245 31067713

[170] Li Y, Altan E, Pilcher C, Hartogensis W, Hecht FM, Deng X, et al. (2020). Semen virome of men with HIV on or off antiretroviral treatment. Aids, 34:827-832. 10.1097/QAD.0000000000002497 32271249

[171] Legoff J, Resche-Rigon M, Bouquet J, Robin M, Naccache SN, Mercier-Delarue S, et al. (2017). The eukaryotic gut virome in hematopoietic stem cell transplantation: new clues in enteric graft-versus-host disease. Nat Med, 23:1080-1085. 10.1038/nm.4380 28759053

[172] Shavaleh R, Kamandi M, Feiz Disfani H, Mansori K, Naseri SN, Rahmani K, et al. (2020). Association between JC virus and colorectal cancer: systematic review and meta-analysis. Infect Dis, 52:152-160. 10.1080/23744235.2019.1692145 31766929

[173] Goel A, Li MS, Nagasaka T, Shin SK, Fuerst F, Ricciardiello L, et al. (2006). Association of JC virus T-antigen expression with the methylator phenotype in sporadic colorectal cancers. Gastroenterol, 130:1950-1961. 10.1053/j.gastro.2006.02.061 16762618

[174] Coelho TR, Gaspar R, Figueiredo P, Mendonça C, Lazo PA, Almeida L (2013). Human JC polyomavirus in normal colorectal mucosa, hyperplastic polyps, sporadic adenomas, and adenocarcinomas in Portugal. J Med Virol, 85:2119-2127. 10.1002/jmv.23705 24009184

[175] Enam S, Del Valle L, Lara C, Gan D-D, Ortiz-Hidalgo C, Palazzo JP, et al. (2002). Association of human polyomavirus JCV with colon cancer: evidence for interaction of viral T-antigen and β-catenin. Cancer Res, 62:7093-7101. 12460931

[176] Ricciardiello L, Baglioni M, Giovannini C, Pariali M, Cenacchi G, Ripalti A, et al. (2003). Induction of chromosomal instability in colonic cells by the human polyomavirus JC virus. Cancer Res, 63:7256-7262. 14612521

[177] Chen H-P, Jiang J-K, Chen C-Y, Chou T-Y, Chen Y-C, Chang Y-T, et al. (2012). Human cytomegalovirus preferentially infects the neoplastic epithelium of colorectal cancer: a quantitative and histological analysis. J Clin Virol, 54:240-244. 10.1016/j.jcv.2012.04.007 22595308

[178] Chen HP, Jiang JK, Lai PY, Chen CY, Chou TY, Chen YC, et al. (2014). Tumoral presence of human cytomegalovirus is associated with shorter disease-free survival in elderly patients with colorectal cancer and higher levels of intratumoral interleukin-17. Clin Microbiol Infect, 20:664-671. 10.1111/1469-0691.12412 24118412

[179] Chen H-P, Jiang J-K, Chan C-H, Teo W-H, Yang C-Y, Chen Y-C, et al. (2015). Genetic polymorphisms of the human cytomegalovirus UL144 gene in colorectal cancer and its association with clinical outcome. J Gen Virol, 96:3613-3623. 10.1099/jgv.0.000308 26450180

[180] Chen H-P, Jiang J-K, Chen C-Y, Yang C-Y, Chen Y-C, Lin C-H, et al. (2016). Identification of human cytomegalovirus in tumour tissues of colorectal cancer and its association with the outcome of non-elderly patients. J Gen Virol, 97:2411-2420. 10.1099/jgv.0.000558 27435237

[181] Teo WH, Chen H-P, Huang JC, Chan Y-J (2017). Human cytomegalovirus infection enhances cell proliferation, migration and upregulation of EMT markers in colorectal cancer-derived stem cell-like cells. Int J Oncol, 51:1415-1426. 10.3892/ijo.2017.4135 PMC5642395 29048611

[183] Harkins L, Volk AL, Samanta M, Mikolaenko I, Britt WJ, Bland KI, et al. (2002). Specific localisation of human cytomegalovirus nucleic acids and proteins in human colorectal cancer. The Lancet, 360:1557-1563. 10.1016/S0140-6736(02)11524-8 12443594

[184] Yang K, Niu J, Zuo T, Sun Y, Xu Z, Tang W, et al. (2021). Alterations in the gut virome in obesity and type 2 diabetes mellitus. Gastroenterol, 161:1257-1269. e1213. 10.1053/j.gastro.2021.06.056 34175280

[185] Faulkner CL, Luo YX, Isaacs S, Rawlinson WD, Craig ME, Kim KW (2021). The virome in early life and childhood and development of islet autoimmunity and type 1 diabetes: A systematic review and meta-analysis of observational studies. Rev Med Virol, 31:1-14. 10.1002/rmv.2209 PMC8518965 33378601

[186] Needell JC, Zipris D (2016). The role of the intestinal microbiome in type 1 diabetes pathogenesis. Curr Diab Rep, 16:1-8. 10.1007/s11892-016-0781-z 27523648

[187] Brown CT, Davis-Richardson AG, Giongo A, Gano KA, Crabb DB, Mukherjee N, et al. (2011). Gut microbiome metagenomics analysis suggests a functional model for the development of autoimmunity for type 1 diabetes. PloS one, 6:e25792. 10.1371/journal.pone.0025792 PMC3197175 22043294

[188] Filippi CM, von Herrath MG (2008). Viral trigger for type 1 diabetes: pros and cons. Diabetes, 57(11):2863. 10.2337/db07-1023 PMC2570378 18971433

[189] Wook Kim K, Allen DW, Briese T, Couper JJ, Barry SC, Colman PG, et al. 2019. Distinct gut virome profile of pregnant women with type 1 diabetes in the ENDIA study. In Open forum infectious diseases: Oxford Univ Press US,. ofz025. 10.1093/ofid/ofz025 PMC6386807 30815502

[190] Zhao G, Vatanen T, Droit L, Park A, Kostic AD, Poon TW, et al. (2017). Intestinal virome changes precede autoimmunity in type I diabetes-susceptible children. Proc Natl Acad Sci, 114:E6166-E6175. 10.1073/pnas.1706359114 PMC5544325 28696303

[191] Cinek O, Kramna L, Odeh R, Alassaf A, Ibekwe MAU, Ahmadov G, et al. (2021). Eukaryotic viruses in the fecal virome at the onset of type 1 diabetes: A study from four geographically distant African and Asian countries. Pediatr Diabetes, 22:558-566. 10.1111/pedi.13207 33786936

[192] Larsen N, Vogensen FK, Van Den Berg FW, Nielsen DS, Andreasen AS, Pedersen BK, et al. (2010). Gut microbiota in human adults with type 2 diabetes differs from non-diabetic adults. PloS One, 5:e9085. 10.1371/journal.pone.0009085 PMC2816710 20140211

[193] Ma Y, You X, Mai G, Tokuyasu T, Liu C (2018). A human gut phage catalog correlates the gut phageome with type 2 diabetes. Microbiome, 6:1-12. 10.1186/s40168-018-0410-y PMC5796561 29391057

[194] Chen Q, Ma X, Li C, Shen Y, Zhu W, Zhang Y, et al. (2021). Enteric phageome alterations in patients with type 2 diabetes. Front Cell infect Microbiol, 10:575084. 10.3389/fcimb.2020.575084 PMC7862107 33552999

[195] Sandoval-Vargas D, Concha-Rubio ND, Navarrete P, Castro M, Medina DA (2021). Obesity intervention resulting in significant changes in the human gut viral composition. Appl Sci, 11:10039.

[196] Hasan MR, Rahman M, Khan T, Saeed A, Sundararaju S, Flores A, et al. (2021). Virome-wide serological profiling reveals association of herpesviruses with obesity. Sci Rep, 11:2562. 10.1038/s41598-021-82213-4 PMC7843976 33510449

[197] Bikel S, López-Leal G, Cornejo-Granados F, Gallardo-Becerra L, García-López R, Sánchez F, et al. (2021). Gut dsDNA virome shows diversity and richness alterations associated with childhood obesity and metabolic syndrome. Iscience, 24:102900. 10.1016/j.isci.2021.102900 PMC8361208 34409269

[198] Yadav H, Jain S, Nagpal R, Marotta F (2016). Increased fecal viral content associated with obesity in mice. World J Diabetes, 7:316. 10.4239/wjd.v7.i15.316 PMC4980638 27555892

[199] Schulfer A, Santiago-Rodriguez TM, Ly M, Borin JM, Chopyk J, Blaser MJ, et al. (2020). Fecal viral community responses to high-fat diet in mice. MSphere, 5:10.1128/msphere.00833-00819. PMC7045389 32102942

[200] Liang W, Feng Z, Rao S, Xiao C, Xue X, Lin Z, et al. (2020). Diarrhoea may be underestimated: a missing link in 2019 novel coronavirus. Gut, 69:1141-1143. 10.1136/gutjnl-2020-320832 32102928

[201] Proal AD, VanElzakker MB (2021). Long COVID or post-acute sequelae of COVID-19 (PASC): an overview of biological factors that may contribute to persistent symptoms. Front Microbiol, 12:698169. 10.3389/fmicb.2021.698169 PMC8260991 34248921

[202] Zuo T, Liu Q, Zhang F, Lui GC-Y, Tso EY, Yeoh YK, et al. (2021). Depicting SARS-CoV-2 faecal viral activity in association with gut microbiota composition in patients with COVID-19. Gut, 70:276-284. 10.1136/gutjnl-2020-322294 PMC7385744 32690600

[203] Albillos A, De Gottardi A, Rescigno M (2020). The gut-liver axis in liver disease: Pathophysiological basis for therapy. J Hepatol, 72:558-577. 10.1016/j.jhep.2019.10.003 31622696

[204] Lang S, Demir M, Martin A, Jiang L, Zhang X, Duan Y, et al. (2020). Intestinal virome signature associated with severity of nonalcoholic fatty liver disease. Gastroenterol, 159:1839-1852. 10.1053/j.gastro.2020.07.005 PMC8404510 32652145

[205] Tarantino G, Citro V, Cataldi M (2021). Findings from studies are congruent with obesity having a viral origin, but what about obesity-related NAFLD? Viruses, 13:1285. 10.3390/v13071285 PMC8310150 34372491

[206] Bajaj JS, Sikaroodi M, Shamsaddini A, Henseler Z, Santiago-Rodriguez T, Acharya C, et al. (2021). Interaction of bacterial metagenome and virome in patients with cirrhosis and hepatic encephalopathy. Gut, 70:1162-1173. 10.1136/gutjnl-2020-322470 32998876

[207] Broecker F, Russo G, Klumpp J, Moelling K (2017). Stable core virome despite variable microbiome after fecal transfer. Gut Microbes, 8:214-220. 10.1080/19490976.2016.1265196 PMC5479397 27935413

[208] Conover KR, Absah I, Ballal S, Brumbaugh D, Cho S, Cardenas MC, et al. (2023). Fecal microbiota transplantation for Clostridioides difficile infection in immunocompromised pediatric patients. J Pediatr Gastroenterol Nutr, 76:440-446. 10.1097/MPG.0000000000003714 PMC10627107 36720105

[209] Fujimoto K, Kimura Y, Allegretti JR, Yamamoto M, Zhang Y-z, Katayama K, et al. (2021). Functional restoration of bacteriomes and viromes by fecal microbiota transplantation. Gastroenterol, 160:2089-2102. e2012. 10.1053/j.gastro.2021.02.013 PMC8684800 33577875

[210] Narula N, Kassam Z, Yuan Y, Colombel J-F, Ponsioen C, Reinisch W, et al. (2017). Systematic review and meta-analysis: fecal microbiota transplantation for treatment of active ulcerative colitis. Inflamm Bowel Dis, 23:1702-1709. 10.1097/MIB.0000000000001228 28906291

[211] Wong SH, Yu J (2019). Gut microbiota in colorectal cancer: mechanisms of action and clinical applications. Nat Rev Gastroenterol Hepatol, 16:690-704. 10.1038/s41575-019-0209-8 31554963

[212] Park R, Umar S, Kasi A (2020). Immunotherapy in colorectal cancer: potential of fecal transplant and microbiota-augmented clinical trials. Curr Colorectal Cancer Rep, 16:81-88. 10.1007/s11888-020-00456-1 PMC7325521 32607098

[213] Wang Y, Wiesnoski DH, Helmink BA, Gopalakrishnan V, Choi K, DuPont HL, et al. (2018). Fecal microbiota transplantation for refractory immune checkpoint inhibitor-associated colitis. Nat Med, 24:1804-1808. 10.1038/s41591-018-0238-9 PMC6322556 30420754

[214] Kazemian N, Kao D, Pakpour S (2021). Fecal microbiota transplantation during and post-COVID-19 pandemic. Int J Mol Sci, 22:3004. 10.3390/ijms22063004 PMC7998826 33809421

[215] Sølbeck Rasmussen T, Junker Mentzel CM, Danielsen MR, Jakobsen RR, Fisker Zachariassen LS, Castro Mejia JL, et al. (2022). Fecal virome transfer improves proliferation of commensal gut Akkermansia muciniphila and unexpectedly enhances the fertility rate in laboratory mice. BioRxiv:2022.2006. 2030.498243. 10.1080/19490976.2023.2208504 PMC10167882 37150906

[216] Bojanova DP, Bordenstein SR (2016). Fecal transplants: what is being transferred? PLoS Biol, 14:e1002503. 10.1371/journal.pbio.1002503 PMC4942072 27404502

[217] Ott SJ, Waetzig GH, Rehman A, Moltzau-Anderson J, Bharti R, Grasis JA, et al. (2017). Efficacy of sterile fecal filtrate transfer for treating patients with Clostridium difficile infection. Gastroenterol, 152:799-811. e797. 10.1053/j.gastro.2016.11.010 27866880

[218] Draper LA, Ryan FJ, Dalmasso M, Casey PG, McCann A, Velayudhan V, et al. (2020). Autochthonous faecal viral transfer (FVT) impacts the murine microbiome after antibiotic perturbation. BMC Biol, 18:1-14. 10.1186/s12915-020-00906-0 PMC7679995 33218339

[219] Brunse A, Deng L, Pan X, Hui Y, Castro-Mejía JL, Kot W, et al. (2022). Fecal filtrate transplantation protects against necrotizing enterocolitis. The ISME journal, 16:686-694. 10.1038/s41396-021-01107-5 PMC8857206 34552194

[220] Lin DM, Koskella B, Lin HC (2017). Phage therapy: An alternative to antibiotics in the age of multi-drug resistance. World [J]. Gastrointest Pharmacol Theraps, 8:162. 10.4292/wjgpt.v8.i3.162 PMC5547374 28828194

[221] Suh GA, Lodise TP, Tamma PD, Knisely JM, Alexander J, Aslam S, et al. (2022). Considerations for the use of phage therapy in clinical practice. Antimicrob Agents Chemother, 66:e02071-02021. 10.1128/aac.02071-21 PMC8923208 35041506

[222] Clavijo V, Baquero D, Hernandez S, Farfan J, Arias J, Arévalo A, et al. (2019). Phage cocktail SalmoFREE® reduces Salmonella on a commercial broiler farm. Poult Sci, 98:5054-5063. 10.3382/ps/pez251 PMC6748741 31073590

[223] Liu N, Lewis C, Zheng W, Fu ZQ (2020). Phage cocktail therapy: multiple ways to suppress pathogenicity. Trends Plant Sci, 25:315-317. 10.1016/j.tplants.2020.01.013 32191865

[224] Farooq T, Hussain MD, Shakeel MT, Tariqjaveed M, Aslam MN, Naqvi SAH, et al. (2022). Deploying viruses against phytobacteria: Potential use of phage cocktails as a multifaceted approach to combat resistant bacterial plant pathogens. Viruses, 14:171. 10.3390/v14020171 PMC8879233 35215763

[225] Galtier M, Sordi LD, Sivignon A, De Vallée A, Maura D, Neut C, et al. (2017). Bacteriophages targeting adherent invasive Escherichia coli strains as a promising new treatment for Crohn’s disease. J Crohns Colitis, 11:840-847. 10.1093/ecco-jcc/jjw224 28130329

[226] Gutiérrez B, Domingo-Calap P. 2020. Phage therapy in gastrointestinal diseases. Microorganisms, 8: 1420. 10.3390/microorganisms8091420 PMC7565598 32947790

[227] Chang RYK, Wallin M, Lin Y, Leung SSY, Wang H, Morales S, et al. (2018). Phage therapy for respiratory infections. Adv Drug Deliv Rev, 133:76-86. 10.1016/j.addr.2018.08.001 PMC6226339 30096336

[228] Wu N, Chen L-K, Zhu T (2022). Phage therapy for secondary bacterial infections with COVID-19. Curr Opin Virol, 52:9-14. 10.1016/j.coviro.2021.11.001 PMC8576063 34800893

[229] Singh AK, Gaur V, Kumar A. 2021. Role of Phage Therapy in COVID-19 Infection: Future Prospects. In Bacteriophages in Therapeutics: IntechOpen.

[230] Lam KN, Spanogiannopoulos P, Soto-Perez P, Alexander M, Nalley MJ, Bisanz JE, et al. (2021). Phage-delivered CRISPR-Cas9 for strain-specific depletion and genomic deletions in the gut microbiome. Cell Rep, 37. 10.1016/j.celrep.2021.109930 PMC8591988 34731631

[231] Zheng D-W, Dong X, Pan P, Chen K-W, Fan J-X, Cheng S-X, et al. (2019). Phage-guided modulation of the gut microbiota of mouse models of colorectal cancer augments their responses to chemotherapy. Nat Biomed Eng, 3:717-728. 10.1038/s41551-019-0423-2 31332342

[232] Selle K, Fletcher JR, Tuson H, Schmitt DS, McMillan L, Vridhambal GS, et al. (2020). In vivo targeting of Clostridioides difficile using phage-delivered CRISPR-Cas3 antimicrobials. MBio, 11:10.1128/mbio.00019-00020. PMC7064742 32156803

[233] Gencay YE, Jasinskytė D, Robert C, Semsey S, Martínez V, Petersen AØ, et al. (2024). Engineered phage with antibacterial CRISPR-Cas selectively reduce E. coli burden in mice. Nat Biotechnol, 42:265-274. 10.1038/s41587-023-01759-y PMC10869271 37142704

[234] Lang JM, Eisen JA, Zivkovic AM (2014). The microbes we eat: abundance and taxonomy of microbes consumed in a day’s worth of meals for three diet types. PeerJ, 2:e659. 10.7717/peerj.659 PMC4266855 25538865

[235] Manrique P, Dills M, Young MJ (2017). The human gut phage community and its implications for health and disease. Viruses, 9:141. 10.3390/v9060141 PMC5490818 28594392

[236] O’Toole PW, Jeffery IB (2015). Gut microbiota and aging. Science, 350:1214-1215. 10.1126/science.aac8469 26785481

